# Identification and Functional Study of Chitin Metabolism and Detoxification-Related Genes in *Glyphodes pyloalis* Walker (Lepidoptera: Pyralidae) Based on Transcriptome Analysis

**DOI:** 10.3390/ijms21051904

**Published:** 2020-03-10

**Authors:** Zuo-min Shao, Yi-jiangcheng Li, Xiao-rui Zhang, Jie Chu, Jia-hui Ma, Zhi-xiang Liu, Jun Wang, Sheng Sheng, Fu-an Wu

**Affiliations:** 1 Jiangsu Key Laboratory of Sericultural Biology and Biotechnology, School of Biotechnology, Jiangsu University of Science and Technology, Zhenjiang 212018, China; shaozuomin23@163.com (Z.-m.S.); 192310019@stu.just.edu.cn (Y.-j.L.); zxr1131286377@sina.com (X.-r.Z.); 18362885591@163.com (J.C.); 18852864112@163.com (Z.-x.L.); wangjun@just.edu.cn (J.W.); 2 Zhenjiang Runshen Sericulture Development Co., Ltd, Zhenjiang 212001, China; JiahuiMa1030@hotmail.com; 3 The Key Laboratory of Silkworm and Mulberry Genetic Improvement, Ministry of Agriculture, Sericultural Research Institute, Chinese Academy of Agricultural Science, Zhenjiang 212018, China

**Keywords:** *Glyphodes pyloalis* Walker, transcriptome analysis, chitin metabolism, detoxifying enzymes, biological control

## Abstract

*Glyphodes pyloalis* Walker (Lepidoptera: Pyralididae) is a serious pest in the sericulture industry, which has caused damage and losses in recent years. With the widespread use of insecticides, the insecticide resistance of *G. pyloalis* has becomes increasingly apparent. In order to find other effective methods to control *G. pyloalis*, this study performed a transcriptome analysis of the midgut, integument, and whole larvae. Transcriptome data were annotated with KEGG and GO, and they have been shown to be of high quality by RT-qPCR. The different significant categories of differentially expressed genes between the midgut and the integument suggested that the transcriptome data could be used for next analysis. With the exception of Dda9 (GpCDA5), 19 genes were involved in chitin metabolism, most of which had close protein–protein interactions. Among them, the expression levels of 11 genes, including *GpCHSA*, *GpCDA1*, *GpCDA2*, *GpCDA4*, *GPCHT1*, *GPCHT2a*, *GPCHT3a*, *GPCHT7*, *GpTre1*, *GpTre2*, and *GpRtv* were higher in the integument than in the midgut, while the expression levels of the last eight genes, including *GpCHSB*, *GpCDA5*, *GpCHT2b*, *GpCHT3b*, *GpCHT-h*, *GpPAGM*, *GpNAGK*, and *GpUAP*, were higher in the midgut than in the integument. Moreover, 282 detoxification-related genes were identified and can be divided into 10 categories, including cytochrome P450, glutathione S-transferase, carboxylesterase, nicotinic acetylcholine receptor, aquaporin, chloride channel, methoprene-tolerant, serine protease inhibitor, sodium channel, and calcium channel. In order to further study the function of chitin metabolism-related genes, dsRNA injection knocked down the expression of *GpCDA1* and *GpCHT3a*, resulting in the significant downregulation of its downstream genes. These results provide an overview of chitin metabolism and detoxification of *G. pyloalis* and lay the foundation for the effective control of this pest in the sericulture industry.

## 1. Introduction

Mulberry leaves are an important diet for silkworms (*Bombyx mori*, Lepidoptera), and they are also used as shade trees in many countries [1]. The leaf is also a delicious food for many pests. *Glyphodes pyloalis* Walker (Lepidoptera: Pyralidae) is one of them, which causes devastating damage to mulberry leaves every year, especially in some developing countries, such as China, India, Pakistan, etc. *G. pyloalis* not only damages sericulture losses in mulberry leaves, but it also spreads different kinds of viruses to silkworms by feeding polluted mulberry leaves, such as Bombyx densoviruses and picornaviruses [2]. To control *G. pyloalis*, pest insecticides have been widely used, which has led to an obvious insecticide resistance in *G. pyloalis*, thereby causing an outbreak of the mulberry moth in recent years [3]. Meanwhile, it induces environmental pollution caused by the intensive use of pesticides. Therefore, there is an urgent need to find other effective and friendly methods to control *G. pyloalis*.

Chitin is a kind of insoluble structural polysaccharide. The function of chitin is different in different species; for example, it forms structural components in the exoskeleton of arthropods or in the cell walls of fungi and yeast [4] and plays a key role in the biocalcifcation process of coralline algae [5,6]. Moreover, chitin is widely present in the exoskeletal and gut linings, and it plays a key role in all stages of insect development. Therefore, chitin has become a metabolic target of selective pest control [7], which consists of many *N*-acetyl-β-d-glucosamine units linked by β-1,4 glycosidic bonds. Chitin from natural sources is a heteropolymer of *N*-acetyl-β-d-glucosamine and glucosamine in different proportions (usually in the range of 5–20%) [8,9]. Chitin synthesis and degradation are dynamic throughout the developmental stages of insects. In the biosynthesis stage, the chitin content of the epidermis and midgut fluctuates as a complex function of the activities of chitin synthase A (CHSA) and chitin synthase B (CHSB), which forms cuticle and peritrophic matrix with the support of chitin deacetylases (CDAs) [10]. Chitinases (CHTs) controls the degradation of chitin into low-molecular-weight chitooligosaccharides, while β-*N*-acetylglucosaminidases (NAGs) can gradually catalyze the deviation of non-reducing terminal β-*N*-acetylglucosamine residues from chitooligosaccharides [9]. A good example is the degradation of the peritrophic matrix and its replacement with a new matrix during the molting stage [11]. Any defective step can disrupt the balance of biosynthesis and chitin degradation, leading to molting defects and mortality, which has also been demonstrated in some other insects using RNAi, such as *Tribolium castaneum* [12], *Locusta migratoria* [13], and *Haemaphysalis longicornis* [14]. Although the metabolic system of chitin is essential for the growth and development of insects, it has not been found in animals and humans [15]. Therefore, it is of great potential value to develop the biological strategies to disrupt the metabolism balance of chitin in agriculture pest control. However, chitin metabolism in *G. pyloalis* is unclear, so a thorough understanding of this pathway will provide many strategies for achieving pest control. 

The long-term use of insecticides has resulted in significant resistance to pests, and understanding its mechanism will help us take some useful strategies to achieve pest control. To be sure, there are two resistant pathways in pests, namely metabolic resistance and target-site resistance [16]. Metabolic resistance depends on the overexpression of three major metabolic detoxification enzymes, including cytochrome P450 (CYP), carboxylesterases (CarEs), and glutathione S-transferases (GSTs) [17]. Target-site resistance is derived from the mutation or modification of genes encoding the target proteins [18,19]. According to transcriptome anakysis, different resistant strains after treatment with propoxur and phoxim identified the resistance-related genes of *G. pyloalis*. After exposure to propoxur, the genes encoding CYP324A19, CYP304F17, CYP6AW1, CYP6AB10, GSTs5, and acetylcholinesterase were significantly upregulated. Moreover, 21 single nucleotide polymorphisms (SNP) have been identified [3]. However, this report is limited to identifying genes that are resistant to propoxur and phoxim treatment and does not fully elucidate its mechanism of detoxification. 

In this study, transcriptome sequencing was used to analyze the midgut, integument, and whole larvae in identifying the genes involved in chitin metabolism and detoxification. Data quality was validated by RT-qPCR. RNAi confirmed the function of interesting genes. Thus, these data will provide genes that can be used for further mechanistic studies of pest control and the detoxification mechanism of *G. pyloalis*.

## 2. Results

### 2.1. Overview of G. pyloalis Transcriptome

There have been few reports on *G. pyloalis*, and there is an urgent need to summarize the data to support further research. Transcriptome has been widely used as an effective technique to obtain a global overview of gene expression levels under different conditions, especially for species without reference genomes. In this study, RNA-Seq technology was used to analyze different tissues of *G. pyloalis*, including the midgut, integument, and whole larvae. In addition, the Illumina HiSeqTM 4000 system is used for RNA sequencing, with a total high-quality reads 37,118 unigenes, an average length of 1029 bp, a maximum length of 55,047 bp, a minimum length of 201 bp, and an N50 value of 1736. If all unigenes are sequenced from long to short and then the total length is added up, when the cumulative fragment length reaches 50% of the total fragment length (the length of all unigenes), the corresponding length of that fragment will be the length of the unigene N50. The quality evaluation of the assembly results can be evaluated from the N50 value (Table 1 and Table S1). The longer the unigene N50 is, the better the assembly quality. Therefore, the results of this study indicate that the transcriptome data has high assembly quality and can be used for further research.

### 2.2. Unigenes Annotation and Classification

The assembled unigenes sequences were annotated by aligning with those deposited in various protein databases using a BLASTX program with a cutoff E-value of 10^−5^, including the NCBI nr database (http://www.ncbi.nlm.nih.gov), KEGG (http://www.genome.jp/kegg), UniProt/Swiss-Prot (http://www.expasy.ch/sprot), GO and COG (http://www.ncbi.nlm.nih.gov/COG). The results show that 20,100 unigenes have annotations, of which the nr database (19,949) contains most of the matched unigenes, and the KEGG contains 18,327 annotation, the COG contains 10,954 and the SwissProt contains 11,631. In the Nr annotation, 5330 (14.33%) unigenes were annotated in *Amyelois transitella*, 2330 (627%) unigenes were annotated in *B. mori*, 1906 (5.13%) unigenes were annotated in *Papilio xuthus*, 1636 (4.41%) unigenes were annotated in *Papilio machaon*, and the last part were annotated in some other species (Figure S1). Unfortunately, the remaining 17,018 unigenes still do not have annotated data. 

### 2.3. The Analysis of KEGG Pathway and GO Annotation between Integument and Midgut

**Figure 1:**
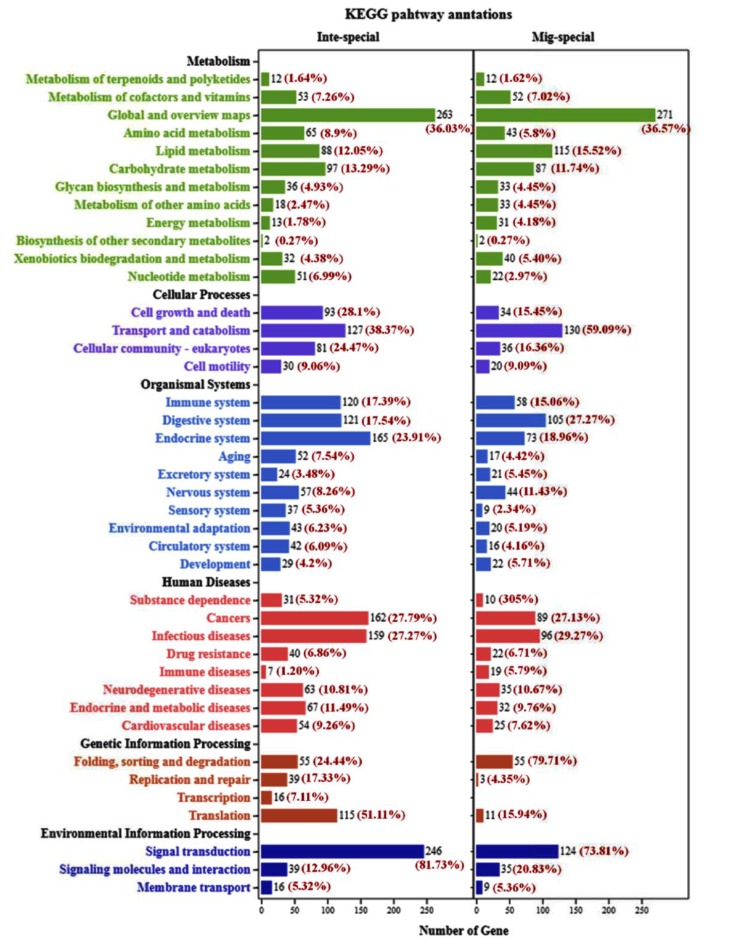
The KEGG analysis of differentially expressed genes between integument and midgut. Genes with RPKM ≥1 and the ratio between integument and midgut ≥2 were selected for comparative analysis.

In order to identify the differences between integument and midgut in the KEGG pathway and the GO annotation, genes with reads per kilobase per million mapped reads (RPKM) ≥1 and the ratio between integument and midgut ≥2 were selected for comparative analysis. A total of 5398 and 3781 genes were obtained and analyzed in the midgut and integument, respectively. In the KEGG pathway, these genes were classified into six pathways, including metabolism, cellular process, organismal systems, human diseases, genetic information processing, and environmental information processing. The genes involved in energy metabolism (4.18%), immune diseases (5.79%), and folding, sorting, and degradation (79.71%) were identified, and the ratio of the midgut was more than twice that of the integument. Otherwise, the genes related to nucleotide metabolism (6.99%), sensory system (5.36%), replication and repair (17.33%), transcription (7.11%), and translation (51.11%) were identified, with a ratio of the integument being more than twice that of the midgut (Figure 1). 

In the classification of GO annotations, genes with RPKM ≥1 and the ratio between integument and midgut ≥2 were classified into three categories, including biological process, molecular function, and cellular component. Genes were classified as cell killing (0.38%), growth (0.38%), multicellular organismal process (2.91%), molecular transducer activity (2.58%), nucleic acid binding transcription factor activity (3.87%), structural molecular activity (4.61%), extracellular matrix (0.28%), supramolecular fiber (0.55%), and synapse (0.83%), were identified with a ratio of the integument being more than twice that of the midgut (Figure S2). In contrast, classification of the genes into the positive regulation of biological process (1.14%), antioxidant activity (0.73%), and membrane (24.03%) were identified with a ratio of the integument being more than twice that of the midgut.

### 2.4. The Validation of Transcriptome Data by RT-qPCR

**Figure 2:**
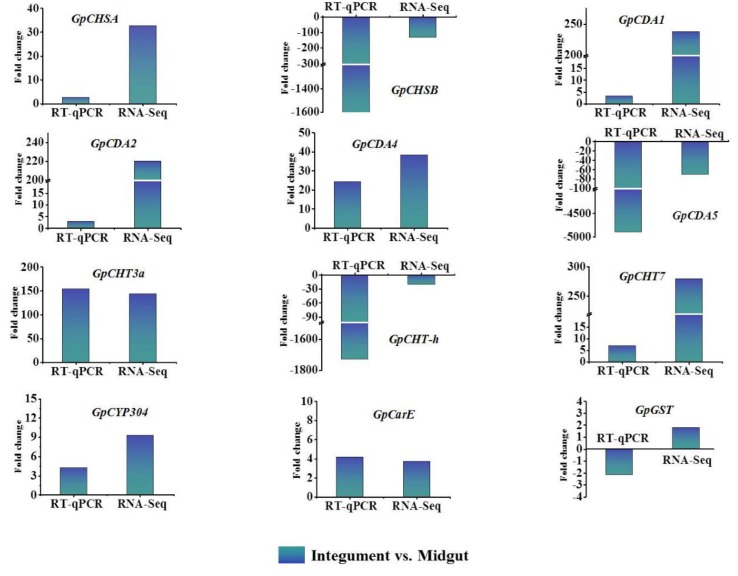
Correlation between gene expression ratios obtained from transcriptome data and RT-qPCR. Data were normalized using *GpRpl32* and expressed as the mean ± standard error of the mean from three independent experiments. The relative expression level was calculated using the 2^−^^△△Ct^ method. The ratios were obtained by comparing unigenes expression levels in integument against midgut. *GpCHSA*, *chitin synthase A*; *GpCHSB*, *chitin synthase B*; *GpCDA1*, *chitin deacetylase 1*; *GpCDA2*, *chitin deacetylase 2*; *GpCDA4*, *chitin deacetylase 4*; *GpCDA5*, *chitin deacetylase 5*; *GpCHT3a*, *chitinase 3a*; *GpCHT-h*, *chitinase-h*; *GpCHT7*, *chitinase 7*; *GpCarE*, *carboxylesterase*; *GpGST*, *glutathione S-transferase*; *GpCYP304*, *cytochrome P450 monooxygenase 304*.

To determine the reliability of unigenes expression levels in transcriptome data, the relative expression levels of 12 randomly selected genes (*GpCHSA*, *GpCDA1*, *GpCDA2*, *GpCDA5*, *GpCHT-h*, *GpCarE*, *GpCDA4*, *GpCHT3a*, *GpCYP304*, *GpCHSB*, *GpCHT7*, *GpGST*) from chitin metabolism-related genes and detoxification-related genes were analyzed in the midgut, integument, and whole larvae by RT-qPCR (Figure 2). The results show that the expression levels of *GpCHSA*, *GpCDA1*, *GpCDA2*, *GpCarE*, *GpCDA4*, *GpCHT3a*, *GpCYP304*, and *GpCHT7* are higher in the integument than in the midgut, which is consistent with the transcriptome data. The expression levels of *GpCDA5a*, *GpCHT-h*, and *GpCHSB* are lower in the integument than in the midgut, which is also consistent with the transcriptome data. However, the expression level of *GpGST* in RT-qPCR is reversed with transcriptome data. Subsequently, the expression level of *GpGST* in different tissues was analyzed (Figure S3), indicating that *GpGST* has a higher expression in the integument than in the midgut. In general, the transcriptome data is satisfactory for further analysis. 

### 2.5. Transcripts Encoding Chitin Metabolism Enzymes

Chitin is a major component of the exoskeleton and peritrophic matrix of insects. The synthesis, deacetylate, and degradation of chitin are dynamic in different developmental stages; hence, chitin plays an essential role in insect growth. This is why chitin can be used as a potential pest control target. As a good example, diflubenzuron (DFB) has been widely reported to interfere with chitin metabolism in pest control [20,21,22]. However, chitin metabolism in *G. pyloalis* is still unclear. In this study, 19 genes encoding chitin metabolism-related enzymes were identified, and BlastX was also used to manually check the GenBank Nr protein database at NCBI to verify whether they are identical to insect chitin metabolism-related genes (Table 2). Meanwhile, the sequences of these genes have been stored in GenBank, and the accession numbers are listed in Table 2. Based on the Nr annotation, these genes may encode chitinases 1, 2a, 2b, 3a, 3b, h, and 7 (CHT1, 2a, 2b, 3a, 3b, h, and 7), chitin synthase A and B (CHSA and CHSB), chitin deacetylases 1, 2, 4, and 5 (CDA1, 2, 4, and 5), *N*-phosphoacetylglucosamine mutase (PAGM), *N*-acetyl-d-glucosamine kinase (NAGK), UDP-*N*-acetylglucosamine pyrophosphorylase (UAP), trehalase 1 and 2 (Tre 1 and Tre 2), and retroactive (Rtv). These results provide potentially valuable data for the control of *G. pyloalis*. Moreover, the expression levels of *GpCHSA*, *GpCDA1*, *GpCDA2*, *GpCDA4*, *GpCHT1*, *GpCHT2a*, *GpCHT3a*, *GpCHT7*, *GpTre1*, *GpTre2*, and *GpRtv* detected by RNA-Seq were significantly higher in the integument than in the midgut. The remaining genes in Table 2, including *GpCHSB*, *GpCDA5*, *GpCHT2b*, *GpCHT3b*, *GpCHT-h*, *GpPAGM*, *GpNAGK*, and *GpUAP*, showed higher expression levels in the midgut. Based on these findings, it can be speculated that genes involved in chitin metabolism have a special localization in the integument and midgut, and they can participate in chitin synthesis and degradation in the two pathways of *G. pyloalis*, respectively. 

**Figure 3:**
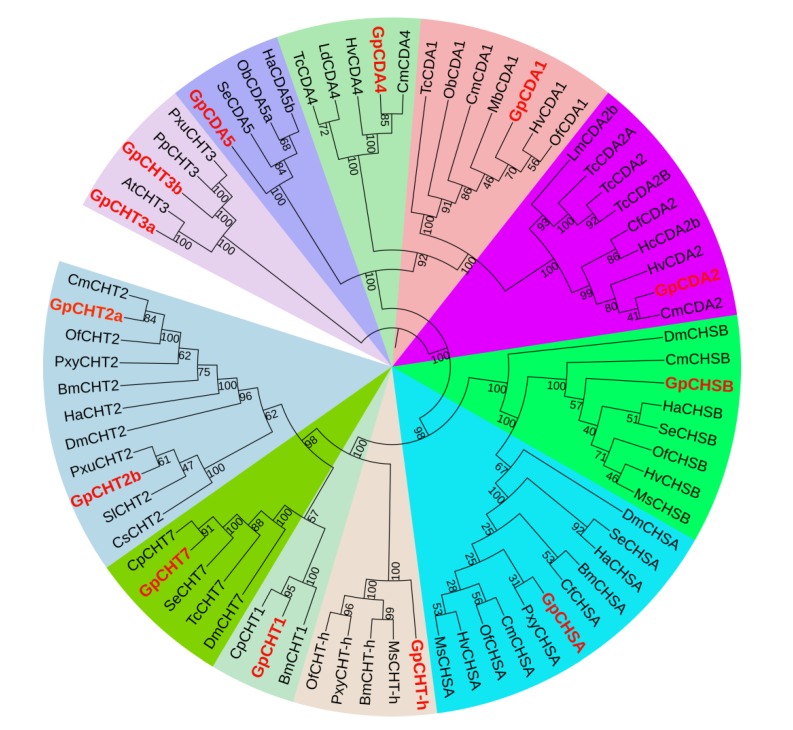
The neighbor-joining tree of chitin metabolism-related enzymes deduced from the coding sequence (CDS) between *G. pyloalis* and other insect species. The tree was generated from multiple alignments using MEGA 6.0 software. The percentages on the branches indicate bootstrap values from 1000 replicates. Chitin metabolism-related enzymes of *G. pyloalis* are indicated in red. *Ostrinia furnacalis* (*Of*), *Cnaphalocrocis medinalis* (*Cm*), *Spodoptera exigua* (*Se*), *Helicoverpa armigera* (*Ha*), *Choristoneura fumiferana* (*Cf*), *Plutella xylostella* (*Pxy*), *Heortia vitessoides* (*Hv*), *Bombyx mori* (*Bm*), *Drosophila melanogaster* (*Dm*), *Manduca sexta* (*Ms*), *Operophtera brumata* (*Ob*), *Hyphantria cunea* (*Hc*), *Locusta migratoria* (*Lm*), *Tribolium castaneum* (*Tc*), *Mamestra brassicae* (*Mb*), *Leptinotarsa decemlineata* (*Ld*), *Conogethes punctiferalis* (*Cp*), *Spodoptera litura* (*Sl*), *Papilio xuthus* (*Pxu*), *Chilo suppressalis* (*Cs*), *Papilio polytes* (*Pp*), and *Amyelois transitella* (*At*).

To analyze the relationship among the major chitin metabolism enzymes identified from transcriptome data, including *GpCHTs*, *GpCDAs*, and *GpCHSs*, the neighbor-joining tree was performed (Figure 3). The deduced CDS of these amino acid sequences from 22 species were constructed using MEGA 6.0. The results show that there is a relatively close relationship between *GpCHT1*, *GpCHT-h*, *GpCHT7*, *GpCHT2a*, and *GpCHT2b*. *GpCHT3a* and *GpCHT3b* are closely related. *GpCHSB* and *GpCHSA* are also closely related. Furthermore, there is a relatively close relationship between *GpCDA1*, *GpCDA2*, *GpCDA4*, and *GpCDA5*. Moreover, *GpCHT2a* has the closest relationship with *CmCHT2*, *GpCHT2b* with *PxuCHT2*, *GpCHT7* with *CpCHT7*, *GpCHT1* with *CpCHT1*, *GpCHSA* with *PxyCHSA*, *GpCHSB* with *HaCHSB*, *GpCDA2* with *CmCDA2*, *GpCDA1* with *HvCDA1*, *GpCDA4* with *CmCDA4*, *GpCDA5* with *SeCDA5*, *GpCHT3a* with *AtCHT3*, and *GpCHT3b* with *PpCHT3*. The results indicate that the functions of these major chitin-metabolizing enzymes in *G. pyloalis* are highly similar to those of other insect species.

### 2.6. Chitin-Metabolizing Enzyme Networks of *G. pyloalis*

**Figure 4:**
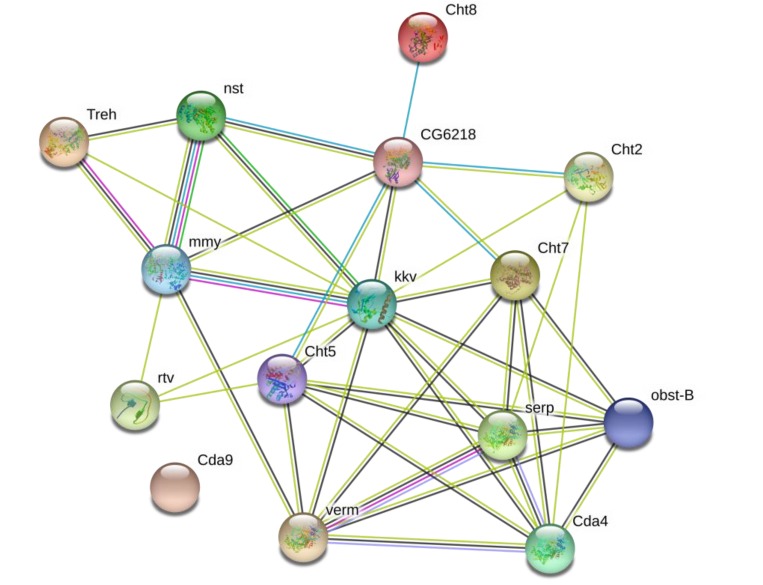
Prediction of protein–protein interaction networks of chitin metabolism-related enzymes based on the STRING website using the *D. melanogaster* database. The homologue of *GpCDA1* in *D. melanogaster* is serp, as GpCDA2 corresponds to verm; GpCDA4 corresponds to Cda4; GpCDA5 corresponds to Cda9; GpCHT1 corresponds to Cht5; GpCHT2a corresponds to Cht2; GpCHT2b and GpCHT-h correspond to Cht8; GpCHT3a corresponds to obst-B; GpCHT3b has no homologue; GpCHT7 corresponds to Cht7; GpCHSA and GpCHSB correspond to kkv; GpNAGK and GpCHSB correspond to CG6218; GpUAP corresponds to CG6218; GpPAGM corresponds to nst; GpRtv corresponds to rtv; GpTre1 and GpTre2 correspond to Treh.

Protein–protein interactions are at the core of the entire interactomics of any living cell and are important for numerous, if not all, biological functions. Therefore, the analysis of the interaction network of enzymes involved in chitin metabolism can provide valuable information for elucidating the entire metabolism pathway of *G. pyloalis*. Herein, 19 candidate chitin metabolism-related enzymes identified from the transcriptome were predicted based on the *D. melanogaster* database using STRING 9.1 online software. Each pair of protein–protein associations is assigned a combined score, which is computed by combining the probabilities from multiple evidences and correcting the probability of randomly observed interactions. The results showed that all proteins, except for Dda9, had a close interaction with a medium confidence (Figure 4). Therefore, it is reasonable to infer that these genes participate in the biosynthesis and degradation of chitin by interacting with each other. 

### 2.7. Identification of Detoxification-Related Genes

The detoxification of pests plays an important role in resistance to insecticide. In addition, it is helpful to control *G. pyloalis* by analyzing its detoxification-related enzymes. Su et al. [3] reported that genes involved in insecticide metabolic processes, including cytochrome P450, glutathione *S*-transferases, and carboxylesterase were identified in the larval midgut of *G. pyloalis* based on the transcriptome analysis of different resistant strains after treatment with propoxur and phoxim; however, these genes responded significantly only to the two insecticides. In this study, RNA-Seq technology was used to sequence the integument, midgut, and whole larvae, and 282 genes involved in detoxification were identified based on the Nr annotation, and then checked on NCBI and an online website (http://geneontology.org/) to further confirm the description.These genes can be divided into 10 categories, including CYP, GST, CarE, nicotinic acetylcholine receptor, aquaporin, chloride channel, methoprene-tolerant, serine protease inhibitor, sodium channel, and calcium channel (Table 3). Among them, CYP (92, 32.6%) accounted for the largest percentage, followed by CarE (59, 21%) and GST (37, 13.1%). Table S3 lists the details of the 10 categories.

### 2.8. The Spatiotemporal Expression Pattern of GpCDA1 and GpCHT3a

**Figure 5:**
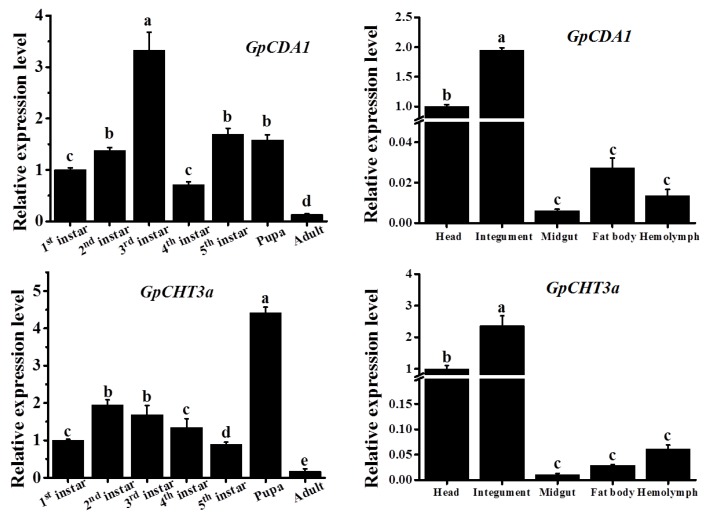
Spatiotemporal expression analysis of *GpCDA1* and *GpCHT3a*. Data were normalized using *GpRpl32* and expressed as the mean ± standard error of the mean from three independent experiments. The relative expression level was calculated using the 2^−^^△△Ct^ method. Differences in the expression levels of each target were compared using a one-way analysis of variance (Systat, Inc., Evanston, IL, USA) with Tukey’s post-hoc test using R version 3.4.0. Significant differences are indicated by different letters, such as, a, b, c (*p* < 0.05).

To preliminarily analyze the specific biological function of *GpCDA1* and *GpCHT3a*, which were selected randomly from the 19 chitin metabolism-related genes, the relative expression levels of the two genes at different developmental stages and tissues were analyzed by RT-qPCR. The results showed that the expression of two genes was detected in all developmental stages, indicating that both genes are involved in chitin metabolism throughout the life of *G. pyloalis* growth. Moreover, the highest level of *GpCDA1* was found in the third instar, while *GpCHT3a* was found in pupa (Figure 5). The lowest expression levels of *GpCDA1* and *GpCHT3a* are all in adults. In different tissues, the relatively higher expression levels of these two genes were in the head and integument, respectively, which may be associated with the high chitin content in the two tissues, suggesting that *GpCDA1* and *GpCHT3a* are involved in the chitin metabolism of *G. pyloalis* integument.

### 2.9. The Analysis of the Function of GpCHT3a in Chitin Metabolism Using dsRNA

**Figure 6:**
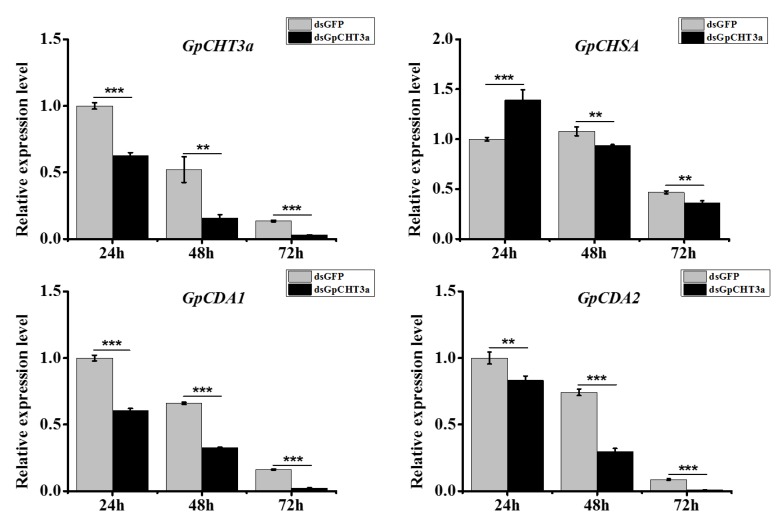
The analysis of the expression of selected downstream genes after the knockdown of *GpCHT3a* at different times. Data were normalized using *GpRpl32* and expressed as the mean ± standard error of the mean from three independent experiments. The relative expression level was calculated using the 2^−^^△△Ct^ method. Differences in the expression levels of each target were compared using a one-way analysis of variance (Systat, Inc., Evanston, IL, USA) with Tukey’s post-hoc test using R version 3.4.0. Significant differences are indicated by asterisks (*p* < 0.05). **, *p* < 0.01; ***, *p* < 0.001.

To knockdown the expression of *GpCHT3a*, a dsRNA targeting the *GpCHT3a* functional domain was designed to knockdown its expression *in vivo*. To maintain the efficiency of RNAi, two targets were selected. Each target with 2.0 μg was mixed and injected with 1.0 μL on the first day of the 5th instar. RT-qPCR was used to analyze the expression of *GpCHT3a* levels at 24 h, 48 h, and 72 h after dsRNA injection. The larvae injected with dsRNA-GFP were used as negative controls. The results showed that the expression of *GpCHT3a* was significantly downregulated after treatment with dsRNA (Figure 6). 

In order to obtain the main regulatory information of *GpCHT3a* on other chitin metabolism-related genes, the expression levels of *GpCHSA*, *GpCDA1*, and *GpCDA2* were analyzed after the RNAi effect of *GpCHT3a* at 24 h, 48 h, and 72 h post injection, respectively. The results showed that the expression levels of the three genes were significantly downregulated after the RNAi effect of *GpCHT3a* (Figure 6), indicating the role of *GpCHT3a* in chitin metabolism. 

### 2.10. The Analysis of the Function of GpCDA1 in Chitin Metabolism Using dsRNA 

**Figure 7:**
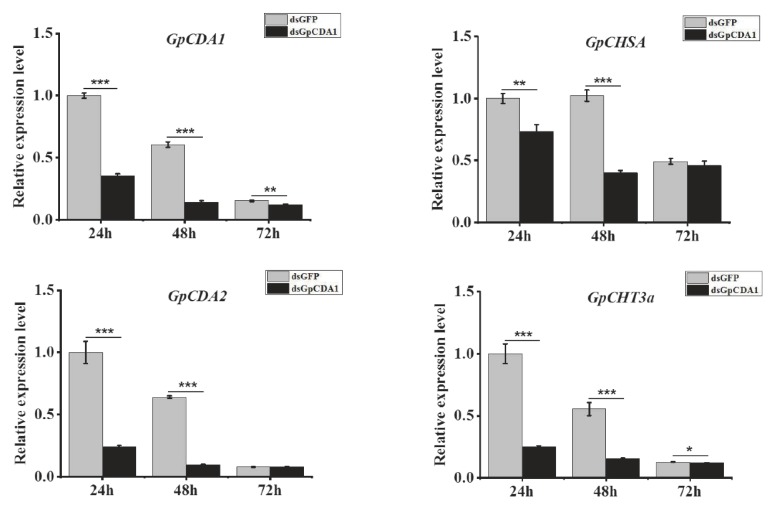
The analysis of the expression of selected downstream genes after the knockdown of *GpCDA1* at different times. Data were normalized using *GpRpl32* and expressed as the mean ± standard error of the mean from three independent experiments. The relative expression level was calculated using the 2^−^^△△Ct^ method. Differences in the expression levels of each target were compared using a one-way analysis of variance (Systat, Inc., Evanston, IL, USA) with Tukey’s post-hoc test using R version 3.4.0. Significant differences are indicated by asterisks (*p* < 0.05). *, *p* < 0.05; **, *p* < 0.01; ***, *p* < 0.001.

As described above, the method used to study the role of *GpCDA1* in chitin metabolism is the same as that of *GpCHT3a*. The effect of RNAi on *GpCDA1* was detected at 24 h, 48 h, and 72 h post injection. The results showed that *GpCDA1* expression was significantly downregulated since 24 h after dsRNA injection. Furthermore, the expression of downstream genes, including *GpCHSA*, *GpCDA2*, and *GpCHT3a*, was also significantly downregulated after 24 h of interference with the dsRNA effect of *GpCDA1* (Figure 7), indicating the role of *GpCDA1* in the chitin metabolism pathway.

## 3. Discussion

With the widespread use of pesticides, the insecticide resistance to pests has become increasingly apparent and very severe. Therefore, there is an urgent need to find other effective and friendly ways to solve such problems. Recently, biological control is one of the effective ways that have been widely studied [23,24]. Chitin exists mainly in the exoskeletal and gut linings of insects, so it is an effective target for pest control. Detoxification enzymes play an important role in insecticide resistance, so identifying these genes can help provide some strategies for solving insecticide resistance problems. However, studies on chitin metabolism and detoxifying enzymes in *G. pyloalis* remain unclear. In this study, the transcriptome analysis of the midgut, integument, and whole larvae of *G. pyloalis* was performed to systematically study the chitin metabolism and detoxifying enzymes. Nineteen (19) genes encoding chitin metabolism-related enzymes were identified, and these genes are mainly involved in the synthesis, deacetylation, and degradation of chitin. Moreover, 282 genes related to detoxification were identified, which can be divided into 10 categories. 

According to reports, chitin metabolism is mainly distributed in the two tissues of midgut and integument [9]. In the midgut, chitin is mainly distributed in the peritrophic matrix, which is an essential component of the insect midgut that plays a vital role in protecting the midgut from mechanical damage by rough food particles, from chemical damage by toxins, and from infection by microorganisms [9]. To identify genes involved in the chitin metabolism pathway, the differences between the midgut and integument of *G. pyloalis* were analyzed. For a more detailed analysis of the characteristics of the two tissues, differentially expressed genes in the two tissues were analyzed based on the KEGG annotation. The results show that there are several significant differences between the midgut and the integument, such as energy metabolism, immune diseases, folding, sorting and degradation, nucleotide metabolism, sensory system, etc. (Figure 1). Moreover, the GO classification analysis also demonstrated some significant differences between the transcriptome data of the two tissues (Figure S2). In general, the analysis of KEGG annotation and GO classification validates that transcriptome data can be used for further analysis. 

Chitin is an essential component of the exoskeleton and the peritrophic matrix of insects, and its biosynthesis and degradation play an important role in the different stages of insect development. In this study, 19 chitin metabolism-related genes were identified, 11 of which showed significantly higher expression levels in the integument compared to the midgut, including *GpCHSA*, *GpCDA1*, *GpCDA2*, *GpCDA4*, *GpCHT1*, *GpCHT2a*, *GpCHT3a*, *GpCHT7*, *GpTre1*, *GpTre2*, and *GpRtv* (Table 2). Chitin synthase (CHS) is an essential enzyme involved in chitin polymerization. The highest expression level of *CHSA* was reported to be in the integument in *Aphis Glycines* and *Acyrthosiphon pisum* [25,26], which is consistent with the expression level of *GpCHSA* (Table 2, Figure 2), indicating its important role in the synthesis of chitin in the integument of *G. pyloalis*. Chitin deacetylases (CDAs) are mainly involved in catalyzing the N-deacetylation of chitin to convert it to deacetylated chitin [23]. It is reported that *CDA1*, *CDA2*, and *CDA4* in several species have relatively high expression levels in the integument than in the midgut, for example *Leptinotarsa decemlineata* [27], *Locusta migratoria* [28,29], and *Manduca sexta* [23], indicating reasonably high expression levels of *GpCDA1*, *GpCDA2*, *GpCDA4* in the integument of *G. pyloalis* (Table 2, Figure 2). Insect chitinases (*CHTs*) play an essential role in the chitin degradation in the integument and peritrophic matrix during the molting process, and it is reported that the expression levels of *PxCHT1*, *PxCHT2a*, *PxCHT3a*, and *PxCHT7* are higher in integument compared to the midgut [30], which is consistent with the results in this study (Table 2, Figure 2). Trehalase is the first enzyme involved in the chitin metabolism pathway, and it plays a critical role in the molting and development of insects [31]. It has been reported that *B. mori trehalase* (*BmTreh*) and *P. xuthus trehalase* (*PxTreh*) can be detected in different tissues at different developmental stages [32]; however, the expression levels of *BmTreh1a*, *BmTreh1b*, *BmTreh2*, *PxTreh1a*, *PxTreh1b*, and *PxTreh2* are quite different with the results of this study (Table 2), which will be further studied in our next step. Retroactive (Rtv) is a vital protein involved in the organization of the newly synthesized procuticular chitin [33]. In *Tribolium castaneum*, TcRtv has been shown to be an essential protein for maintaining normal cuticle architecture [34], indicating the reasonably higher expression levels of *GpTre1* and *GpTre2* in the integument of *G. pyloalis* than in the midgut (Table 2, Figure 2). 

In addition, the remaining eight out of the 19 chitin metabolism-related genes had higher expression levels in the midgut than in the integument, including *GpCHSB*, *GpCDA5*, *GpCHT2b*, *GpCHT3b*, *GpCHT-h*, *GpPAGM*, *GpNAGK*, and *GpUAP* (Table 2). The study of CHS has been widely reported, and the expression of *Locusta migratoria CHS2* is mainly distributed in the midgut [14], which is consistent with the expression of *GpCHSB* detected in this study (Table 2, Figure 2). The relatively high expression level of *GpCDA5* in the midgut is consistent with *HcCDA5* in *Hyphantria cunea*, which is mainly expressed in the midgut of *H. cunea* larvae [35]. GpCHT2b and GpCHT3b showed a highly evolutionary relationship with CHT2 and CHT3 of other species (Figure 3), respectively, indicating that they have played a role in chitin degradation in *G. pyloalis*. The expression levels of *PxCHT-h* and *MsCHT-h* were [23,30] detected and highly expressed in the midgut of *Plutella xylostella* and *Manduca sexta*, which is the same as the results of this study (Table 2). Degraded chitooligosaccharides will be recycled back to the biosynthesis pathway with the help of GpPAGM, GpNAGK and GpUAP, which has also been validated in other species [36,37].

To further validate the results of the transcriptome data and primarily explore the metabolic pathway of chitin, the characterization and function of *GpCDA1* and *GpCHT3a* were studied. At different developmental stages, the highest expression level of *GpCDA1* and *GpCHT3a* was in the 3rd instar and pupa (Figure 5), respectively, which may be related to the degradation and re-synthesis of chitin in the cuticle. In different tissues, the highest expression level of *GpCHT3a* is located in the integument (Figure 5), which further validates the transcriptome data (Table 2). Although the metabolic pathway of chitin has been reported in other species [38], the regulation of the up and downstream genes in *G. pyloalis* has not yet been clarified. To obtain the primary regulatory information of *GpCDA1* and *GpCHT3a* on other chitin metabolism-related genes, the expression levels of *GpCHSA*, *GpCDA2*, *GpCDA1*, and *GpCHT3a* were analyzed after the RNAi effect of *GpCDA1* and *GpCHT3a* at 24 h, 48 h, and 72 h post injection, respectively. The results showed that the expression of these downstream genes was significantly downregulated after the knockdown of *GpCHT3a* and *GpCDA1* at 24 h, 48 h, and 72 h (Figure 6 and Figure 7) and the downregulation of *GpCHT3a* and *GpCDA1* after RNAi against each gene, indicating their regulational relationship in the chitin metabolism pathway.

**Figure 8:**
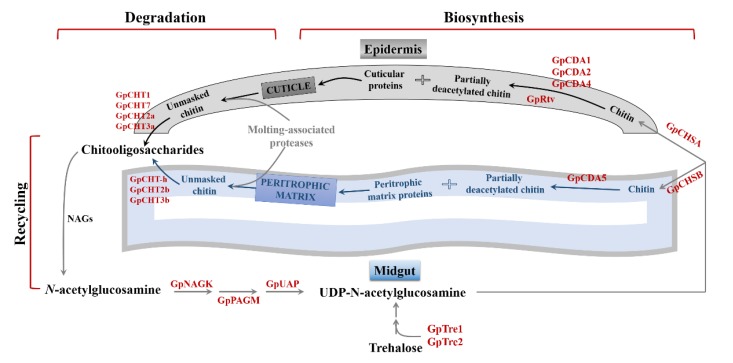
The hypothesis of the main processes, key enzymes, and proteins involved in chitin metabolism in *G. pyloalis*. Abbreviations: Tre1 and Tre2, trehalase 1 and 2; PAGM, N-phosphoacetylglucosamine mutase; CHSA and CHSB, chitin synthase A and B; CDA1, 2, 4, and 5, chitin deacetylase1, 2, 4, and 5; Rtv, retroactive; CHT1, 2a, 2b,3a,3b, 7, and h, chitinase 1, 2a, 2b,3a,3b, 7, and h; NAGK, *N*-acetyl-d-glucosamine kinase; UAP, UDP-*N*-acetylglucosamine pyrophosphorylase.

Based on the data shown in this study and in combination with reference materials related to chitin-metabolizing enzymes, the chitin metabolism pathway in *G. pyloalis* were hypothesized as follows. There are two major pathways involved in the chitin metabolism of *G. pyloalis*: midgut and integument. During the growth of *G. pyloalis*, the trehalose became fragmented and fluxed in the chitin biosynthetic pathway with the help of GpTre 1 and GpTre 2. Chitin was synthesized under the regulation of GpCHSA and GpCHSB. GpCHSB is mainly involved in regulating UDP-*N*-acetylglucosamine flux in the midgut pathway, while GpCHSA regulates its flux in the integument. In the integument, the newly synthesized chitin was modified into partially deacetylated chitin with the help of GpCDA1, GpCDA2, and GpCDA4, and the main component of the cuticle was formatted after the partially deacetylated chitin was combined with cuticular proteins. In the midgut, only GpCDA5 was used to modify the newly synthesized chitin to partially deacetylated chitin, and the main component of peritrophic matrix was formed after the partially deacetylated chitin was combined with peritrophic matrix proteins. During the metamorphosis stage, the cuticle and the peritrophic matrix are degraded into unmasked chitin under the regulation of molting-related proteases. In the integument, the unmasked chitin is degraded in chitooligosaccharides with the aid of GpCHT1, GpCHT7, GpCHT2a, and GpCHT3a, and this progress is controlled under GpCHT-h, GpCHT2b, and GpCHT3b in the midgut. Chitooligosaccharides will be recycled along with several other enzymes, including GpPAGM, GpNAGK, and GpUAP (Figure 8). Finally, this hypothesis will be validated in detail in our subsequent study.

## 4. Materials and Methods

### 4.1. G. pyloalis Rearing and Sample Preparation

*G. pyloalis* larvae were collected in 2014 from mulberry orchards near the School of Biotechnology, Jiangsu University of Science and Technology in Zhenjiang, China and maintained in our larboratory until now. *G. pyloalis* is a five-instar insect, which has a green body as larva, brown pupa, and pale brown spots on adults. It feeds only on the mesophyll of mulberry leaves by secreting fine threads to fold the leaf in the 4th and 5th instar. The larvae were reared with fresh mulberry leaves at 25 ± 1 °C, 60–70% relative humidity, and a 12-h day/night cycle. Transparent plastic boxes covered with a muslin cloth were used for aeration. Fresh mulberry leaves were put in boxes for adult oviposition. The hatched eggs were transferred to another clean box with the help of a soft brush. The humid cotton was placed in a box to maintain moisture.

*G. pyloalis* was selected for sample preparation on the second day of the 5th instar. Thirty (30) midgut, integument, and whole larvae were mixed together to minimize individual genetic differences. All samples were immediately frozen in liquid nitrogen and stored at −80 °C until use.

### 4.2. RNA Extraction and Quality Analysis

According to the manufacturer’s instructions, the total RNA was extracted from the midgut, integument, and whole larvae of *G. pyloalis* were analyzed using TRIzol Reagent (Invitrogen, New York, NY, USA). A NanoDrop 2000 spectrophotometer (Thermo Fisher Scientific, New York, NY, USA) was used to quantify RNA concentration and purity. RNA integrity was analyzed using 1% agarose gel electrophoresis. According to the manufacturer’s instructions, the first strand cDNA was synthesized using an RT reagent kit (Takara Biotechnology Co. Ltd., Dalian, China).

### 4.3. Library Construction, Sequencing, and Assembly

mRNA isolated from total RNA with Oligo (dT) beads was broken into short fragments using fragment buffer. The short fragments were used as tampers to synthesize the first-strand cDNA with a random primer, and then DNA polymerase I was mixed with RNase H, dNTP, and buffer solution to synthesize the complementary strand, which was performed by Guangzhou Genedenovo Biotechnology Co., Ltd (Guangzhou, China). cDNA libraries were prepared and sequenced using standard Illumina methods and protocols. Typically, the end-repaired cDNA fragment was used to purify the 1.8× Agencourt AMPure XP Beads, and this was mixed with sequences of Illumina adapters. The ligation mixture was separated via agarose gel electrophoresis. After PCR amplification, Gene Denovo Biotechnology Co. (Guangzhou, China) sequenced the prepared cDNA libraries on Illumina HiSeqTM 4000 (Illumina, San Diego, CA, USA).

To meet the needs of further analysis, clean and high-quality reads are essential. There are several processing steps to clean up dirty reads: (1) removing read adapters, (2) the removal of unknown nucleotides (ratio ≥ 10%), (3) deleting low-quality reads with a basic mass value Q ≤ 20, accounting for more than 40% of the reads, and (4) obtaining clean reads. 

The clean assembly was performed in accordance with previous reports [39]. De novo transcriptome assembly was adopted using the Trinity short reads assembling program. Detailed parameters (kmer size = 31, min kmer cov = 12; all other non-important parameters are default values) were configured to perform the auto-assembly. According to previous reports, Bowtie2 short reads alignment software (MATLAB, Natick, MA, USA) (Parameters are the default parameters) was used to align the clean reads with reference sequences to obtain an alignment rate [40]. 

### 4.4. Functional Annotation

The BLASTx program (http://www.ncbi.nlm.nih.gov/BLAST/) used for protein functional annotation has an *E*-value threshold of 1 × 10^5^, which follows the priority principle of the National Center for Biotechnology Information (NCBI) non-redundant protein (Nr) database (http://www.ncbi.nlm.nih.gov). The pathway annotation uses the KEGG database (http://www.genome.jp/kegg). In addition, Cluster of Orthologous Groups of proteins (COG/KOG) of proteins functional annotation used the COG/KOG database (http://www.ncbi.nlm. nih.gov/COG). Gene Ontology (GO) annotations, which include molecular functions, biological processes, and cellular components, were obtained using the Blast2GO program (https://www.blast2go.com/) [41,42]. The Swiss-Prot protein database (http://www.expasy.ch/sprot) was also annotated, and the best alignment results were selected as functional annotations. Further, the coding region of unigenes was predicted by ESTScan software [43] as a result of its non-comparison with the above-mentioned libraries, thereby obtaining the coding region of the nucleic acid sequence (sequence direction 50- >30) and amino acid sequence. 

### 4.5. Real-Time Quantitative PCR (RT-qPCR)

In this study, quantitative reverse transcription PCR (RT-qPCR) was adopted to validate transcriptome data and analyze the expression levels of genes of interest. Table 4 shows all primers, in which 15-μL reaction mixtures were prepared with 1.5 μL of cDNA, 7.5 μL of TB Green Fast qPCR Mix (Takara Biotechnology Co. Ltd., Dalian, China), 0.6 μL of each gene-specific primer (0.4 μM), and 0.3 uL of ROX Reference Dye II and 4.5 μL ddH_2_O. The reactions were performed using the QuantStudio™ Real-Time System (Thermo Fisher Scientific, Applied Biosystems, New York, NY, USA). The thermal cycling program consists of 95 °C for 4 min, 40 cycles at 95 °C for 15 s, and 60 °C for 31 s. All reactions were performed in triplicate. The 2^−^^△△Ct^ method was used to calculate the relative expression levels based on the protocol described by Livak et al. [44]. *G. pyloalis ribosomal protein L32* (*GpRpl32*) was adopted as the reference standard gene [3]. Statistical analysis was performed using the R version 3.4.0. The triplicate data in different groups were analyzed by one-way ANOVA with Tukey’s post-hoc test.

### 4.6. Bioinformatics Analysis

The sequence of cDNA and the derived proteins were analyzed using DNAMAN 8.0 software (Lynnon Corporation, Quebec, Canada). In addition, the ortholog sequence was developed using the BLASTP tool (http://www.ncbi.nlm.nih.gov/). The amino acid sequences in different species were aligned with the MUSCLE module using the MEGA6.0 software, while the best DNA/Protein model was first calculated using the “Find best DNA/Protein Model program” in MEGA6.0 software. A phylogenetic tree was generated using the neighbor-joining method with 1000 bootstrap replications. The chitin metabolism-related genes derived from 22 insect species were used to generate trees, and their GenBank IDs are listed in Table S2. 

The protein–protein interactions (PPIs) of chitin metabolism-related enzymes were analyzed through the STRING online website (http://string-db.org/). Due to the incomplete proteomic information of *G. pyloalis* in STRING, the PPI network was built using the *Drosophila melanogaster* database, which was very well.

### 4.7. The Synthesis of dsRNA and Quality Analysis

To analyze the functions of *GpCDA1* and *GpCHT3a* involved in chitin metabolism, two special targets of the functional domain of each gene were selected. dsRNA oligos were designed and sent to Sangon Biotechnology in China for synthesis (Table 5). dsRNA was synthesized using an *In Vitro* Transcription T7 Kit (for siRNA Synthesis) (Takara Biotechnology Co. Ltd., Dalian, China) in accordance with the manufacturer’s instructions. The NanoDrop 2000 spectrophotometer was used to detect the concentration and purity of dsRNA. The RNA quality was examined by 3% agarose gel electrophoresis, and subsequently stored at −80 °C until use.

## Figures and Tables

**Table 1:** Summary statistics of *G. pyloalis* genes based on RNA-seq sequencing.

Sample	Raw Data (bp)	Clean Data (bp)	Q20 (%)	Q30 (%)	GC Content (%)
Inte-1	7,986,373,500	7,947,476,345	97.65%	93.45%	52.98%
Inte-2	7,641,577,800	7,605,711,075	97.88%	94.01%	52.77%
Inte-3	7,992,333,000	7,956,556,516	97.83%	93.86%	53.66%
Midg-1	9,036,411,000	8,924,294,465	97.06%	92.52%	53.10%
Midg-2	7,794,233,100	7,716,963,009	97.36%	93.11%	52.75%
Midg-3	7,476,890,700	7,412,795,894	97.38%	93.10%	52.58%
Larv-1	5,629,523,700	5,593,818,739	97.84%	93.94%	52.24%
Larv-2	6,382,601,400	6,344,754,317	97.61%	93.40%	51.73%
Larv-3	7,023,010,500	6,978,558,841	97.56%	93.33%	51.66%

Inte represents integument, Midg represents midgut, Larv represents whole larva.

**Table 2:** Identification of major enzyme genes involved in the chitin metabolism of *G. pyloalis*.

Gene Name	Accession No.	BLASTX Best Hit				Integument (RPKM)	Midgut (RPKM)	Larva (RPKM)
		Species	Gene Description	Accession No.	E-value			
*GpCHS A*	MN915086	*Ostrinia furnacalis*	Chitin synthase A	ACB13821.1	0.0	24.39	0.75	5.66
*GpCHS B*	MN915087	*Cnaphalocrocis medinalis*	Chitin synthase B	AJG44539.1	0.0	1.99	265.97	88.34
*GpCDA1*	MN915088	*Ostrinia furnacalis*	Chitin deacetylase 1	QDZ05988.1	0.0	899.05	3.79	226.69
*GpCDA2*	MN915096	*Choristoneura fumiferana*	CDA2 isoform B	AGT28749.1	0.0	558.26	2.54	144.88
*GpCDA4*	MN915090	*Cnaphalocrocis medinalis*	Chitin deacetylase 4	AJG44548.1	0.0	31.24	0.82	7.73
*GpCDA5*	MN915091	*Operophtera brumata*	Chitin deacetylase 5a, partial	KOB56571.1	6e-152	70.44	4961.48	1818.85
*GpCHT1*	MN915094	*Conogethes punctiferalis*	Chitinase 1	ASM94206.1	0.0	10.11	0.18	2.52
*GpCHT2a*	MN915093	*Cnaphalocrocis medinalis*	Chitinase 2	AJG44542.1	0.0	20.32	7.39	9.71
*GpCHT2b*	MN915098	*Amyelois transitella*	PREDICTED: probable chitinase 2	XP_013190968.1	0.0	0.04	30.74	15.47
*GpCHT3a*	MN915089	*Amyelois transitella*	PREDICTED: probable chitinase 3	XP_013183423.1	4e-169	249.83	1.75	61.94
*GpCHT3b*	MN915097	*Papilio polytes*	PREDICTED: probable chitinase 3	KPI96666.1	2e-47	0.10	236.62	79.34
*GpCHT-h*	MN915092	*Samia cynthia*	Chitinase	BAE16586.1	0.0	2.63	55.59	19.26
*GpCHT7*	MN915095	*Conogethes punctiferalis*	Chitinase 7	ASM94207.1	0.0	49.42	0.18	11.75
*GpPAGM*	MN915104	*Cnaphalocrocis medinalis*	*N*-phosphoacetylglucosamine mutase	AJG44540.1	0.0	7.42	22.07	10.55
*GpNAGK*	MN915100	*Omphisa fuscidentalis*	*N*-acetyl-d-glucosamine kinase	KPJ05170.1	2e-159	2.94	9.39	4.51
*GpUAP*	MN915103	*Cnaphalocrocis medinalis*	UDP-*N*-acetylglucosamine pyrophosphorylase	AKO90063.1	0.0	15.16	142.19	45.74
*GpTre1*	MN915101	*Omphisa fuscidentalis*	Soluble trehalase	ANY30160.1	0.0	71.42	39.16	30.21
*GpTre2*	MN915102	*Omphisa fuscidentalis*	Trehalase-2	ABO20845.1	0.0	27.55	21.91	17.49
*GpRtv*	MN915099	*Papilio xuthus*	retroactive	BAM18479.1	3e-61	9.45	1.35	4.69

**Table 3:** Unigenes potentially involved in the detoxification of *G. Pyloalis.*

Genes	Number (Percentage)
Cytochrome P450	92 (32.6%)
Glutathione *S*-transferase	37 (13.1%)
Carboxylesterase	59 (21%)
Nicotinic acetylcholine receptor	7 (2.5%)
Aquaporin	19 (6.7%)
Chloride channel	3 (1.1%)
Methoprene-tolerant protein	2 (0.7%)
Serine protease inhibitor	22 (7.8%)
Sodium channel	11 (3.9%)
Calcium channel	30 (10.6%)

**Table 4:** Primers used in quantitative reverse transcription PCR (RT-qPCR).

Gene Names	Forward Primer (5′-3′)	Reverse Primer (5′-3′)
*GpCHSA*	TACGCTTTCCACATCACCGC	ACGGGCCTTCTCTTCCTTGT
*GpCHSB*	ACTTGGCTTTGGGCAGCTTT	GGTCCCTCGTCAACGCATTT
*GpCDA1*	TTCAAGCCATTCGCTGTCCC	CCAGGAAGCCATCTTGGCAG
*GpCDA5*	GTGCTTCCCTCCTAACACGC	CCCTTTCGATGGCAGGGTTC
*GpCDA2*	TTGGTGTGCGTGCTCCTTAC	TATGGGCGTTACCGTTGCAC
*GpCHT-h*	GCGACCCTTACAGAGGCAAC	TTTTCGCTTCACCGCATCGT
*GpCHT7*	GGAGGAGTCTGTGGTGGGAA	ACGCTACTGAGGCCCAATCT
*GpCDA4*	GCCCTATACCAACAACGCCC	AGAAAGTCCCGCGTATGGGA
*GpCHT3a*	TTCAACGACTACAGCCCCGA	GAGAAGTAGCCGTTCAGGCG
*GpCYP304*	GGCCGTGAATGGACCCAAAT	GCCACGTGCCAACTCATACA
*GpGST*	GGGCACTCAACCTGAACCTC	TCGCGTATAGGGAGCCGATG
*GpCarE*	ATTGTGCGCATAGAGCAGGG	ACGGCATCCAAAACCGACAA
*GpRpl32*	CGATCACCTTCCGCTTCT	TGCTACCCAATGGCTTCC

**Table 5:** Primers used to synthesize dsRNA.

Primer Names	Sequence (5′-3′)
GpCDA1-1-Olig-1	GATCACTAATACGACTCACTATAGGGGCAGACTTGTGACTGGAAATT
GpCDA1-1-Olig-2	AATTTCCAGTCACAAGTCTGCCCCTATAGTGAGTCGTATTAGTGATC
GpCDA1-1-Olig-3	AAGCAGACTTGTGACTGGAAACCCTATAGTGAGTCGTATTAGTGATC
GpCDA1-1-Olig-4	GATCACTAATACGACTCACTATAGGGTTTCCAGTCACAAGTCTGCTT
GpCDA1-2-Olig-1	GATCACTAATACGACTCACTATAGGGGCGACATTAAAGCCACCTTTT
GpCDA1-2-Olig-2	AAAAGGTGGCTTTAATGTCGCCCCTATAGTGAGTCGTATTAGTGATC
GpCDA1-2-Olig-3	AAGCGACATTAAAGCCACCTTCCCTATAGTGAGTCGTATTAGTGATC
GpCDA1-2-Olig-4	GATCACTAATACGACTCACTATAGGGAAGGTGGCTTTAATGTCGCTT
GpCHT3a-1-Olig-1	GATCACTAATACGACTCACTATAGGGGCAGTGCGACAAGTACTATTT
GpCHT3a-1-Olig-2	AAATAGTACTTGTCGCACTGCCCCTATAGTGAGTCGTATTAGTGATC
GpCHT3a-1-Olig-3	AAGCAGTGCGACAAGTACTATCCCTATAGTGAGTCGTATTAGTGATC
GpCHT3a-1-Olig-4	GATCACTAATACGACTCACTATAGGGATAGTACTTGTCGCACTGCTT
GpCHT3a-2-Olig-1	GATCACTAATACGACTCACTATAGGGCCCTTCAACATCGACTGTTTT
GpCHT3a-2-Olig-2	AAAACAGTCGATGTTGAAGGGCCCTATAGTGAGTCGTATTAGTGATC
GpCHT3a-2-Olig-3	AACCCTTCAACATCGACTGTTCCCTATAGTGAGTCGTATTAGTGATC
GpCHT3a-2-Olig-4	GATCACTAATACGACTCACTATAGGGAACAGTCGATGTTGAAGGGTT

## Supplementary Materials

Supplementary materials can be found at https://www.mdpi.com/1422-0067/21/5/1904/s1.
