# Supplementary material for: Identification and Functional Study of Chitin Metabolism and Detoxification-Related Genes in Glyphodes pyloalis Walker (Lepidoptera: Pyralidae) Based on Transcriptome Analysis
Source: Int J Mol Sci. 2020 Mar 10;21(5):1904. doi: 10.3390/ijms21051904 (PMC7084822; doi:10.3390/ijms21051904)
Supplement: Supplementary file 1 [file ijms-21-01904-s001.pdf]

Supplementary materials

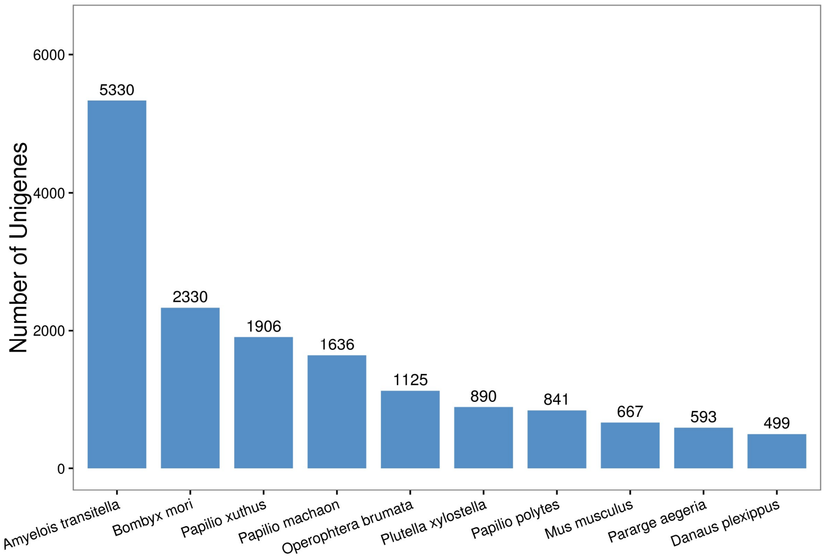

Figure S1. The Nr annotation of the transcriptome data.

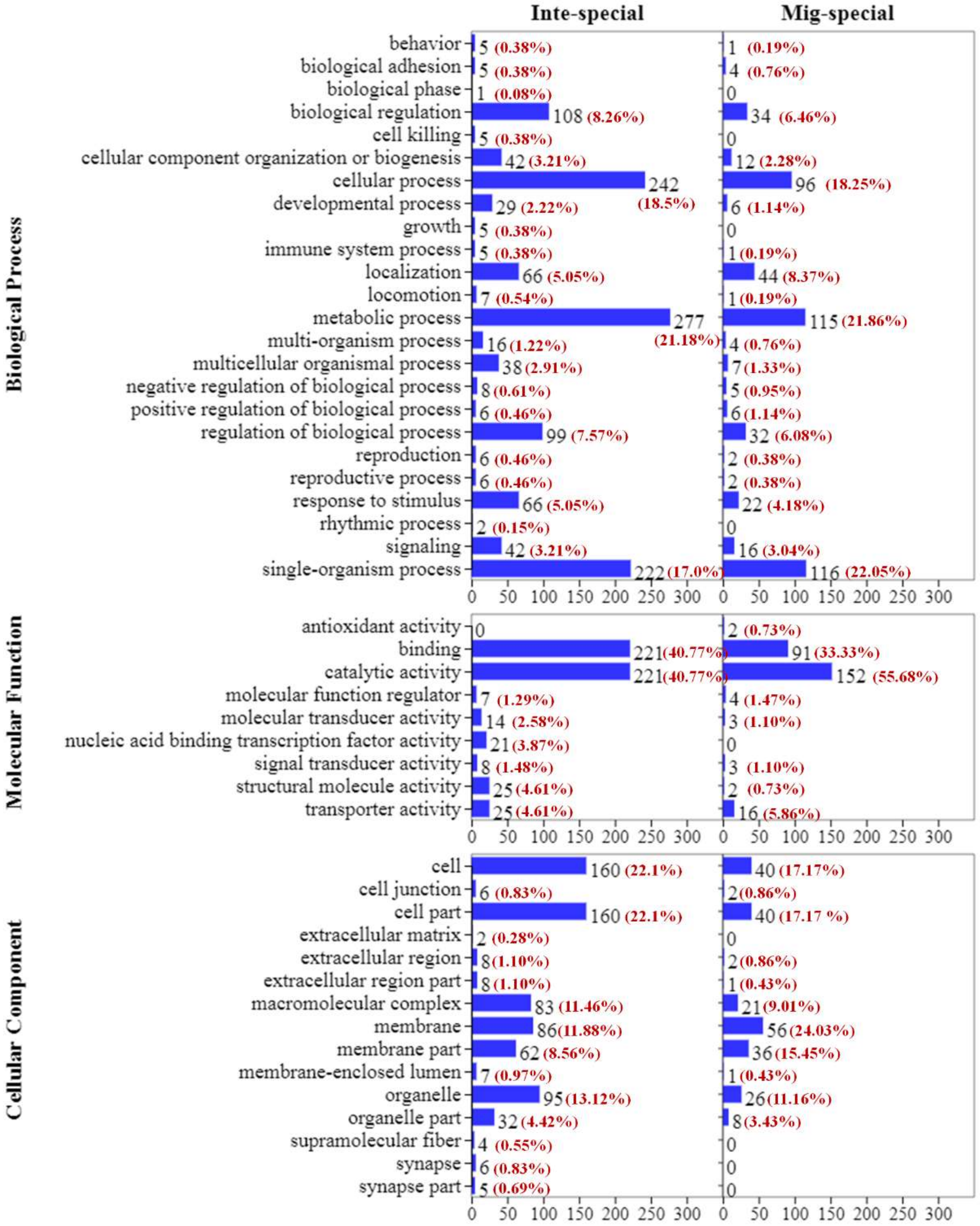

Figure S2. The GO analysis of differentially expressed genes between the integument and the midgut.

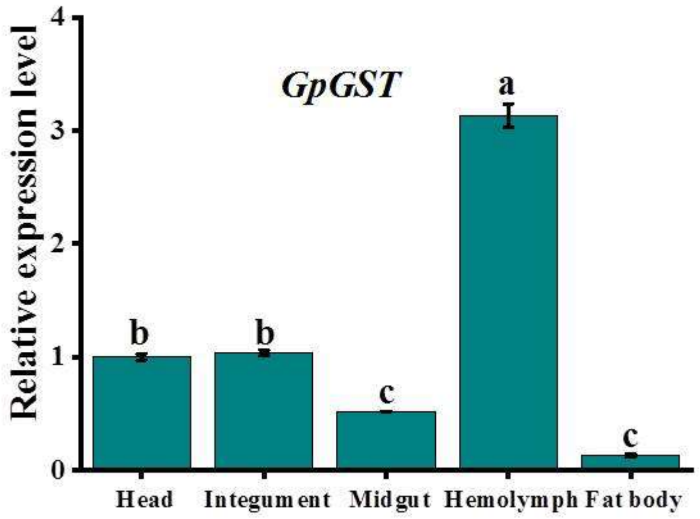

Figure S3. The expression level analysis of GpGST in different tissues using RT-qPCR.

Table S1. Assembly quality statistics of transcriptome data

Genes Num	GC percentage	N50 number	N50 length	Max length	Min length	Average length	Total assembled bases
37,118	43.63%	6304	1736	55,047	201	1029	38,195,251

Table S2. The detail information of chitin metabolism-related enzymes genes involved in phylogenetic analysis

Genes name	GenBank No.	Species	Phylogenetic group
OfCHSA	ACF53745.1	*Ostrinia furnacalis*	Lepidoptera
CmCHSA	AJG44538.1	*Cnaphalocrocis medinalis*	Lepidoptera
SeCHSA	AAZ03545.1	*Spodoptera exigua*	Lepidoptera
HaCHSA	QEQ50332.1	*Helicoverpa armigera*	Lepidoptera
CfCHSA	ACD84882.1	*Choristoneura fumiferana*	Lepidoptera
PxyCHSA	API61827.1	*Plutella xylostella*	Lepidoptera
HvCHSA	AZQ19982.1	*Heortia vitessoides*	Lepidoptera
BmCHSA	AFB83705.1	*Bombyx mori*	Lepidoptera
DmCHSA	NP_524233.1	*Drosophila melanogaster*	Diptera
MsCHSA	AAL38051.2	*Manduca sexta*	Lepidoptera
CmCHSB	AJG44539.1	*Cnaphalocrocis medinalis*	Lepidoptera
OfCHSB	ABB97082.1	*Ostrinia furnacalis*	Lepidoptera
HvCHSB	AZQ19981.1	*Heortia vitessoides*	Lepidoptera
HaCHSB	AKZ08595.1	*Helicoverpa armigera*	Lepidoptera
MsCHSB	AAX20091.1	*Manduca sexta*	Lepidoptera
SeCHSB	ABI96087.1	*Spodoptera exigua*	Lepidoptera
HaCDA5b	ADB43612.1	*Helicoverpa armigera*	Lepidoptera

SeCDA5	AMY98414.1	*Spodoptera exigua*	Lepidoptera
ObCDA5a	KOB56571.1	*Operophtera brumata*	Lepidoptera
CfCDA2	AGT28749.1	*Choristoneura fumiferana*	Lepidoptera
HcCDA2b	AOS49617.1	*Hyphantria cunea*	Lepidoptera
CmCDA2	AJG44547.1	*Cnaphalocrocis medinalis*	Lepidoptera
HvCDA2	QDZ05989.1	*Heortia vitessoides*	Lepidoptera
LmCDA2b	ANA57445.1	*Locusta migratoria*	Lepidoptera
TcCDA2A	NP_001096047.1	*Tribolium castaneum*	Coleoptera
TcCDA2B	NP_001116303.1	*Tribolium castaneum*	Coleoptera
HvCDA1	QDZ05988.1	*Heortia vitessoides*	Lepidoptera
OfCDA1	AKJ26157.1	*Ostrinia furnacalis*	Lepidoptera
CmCDA1	AJG44549.1	*Cnaphalocrocis medinalis*	Lepidoptera
ObCDA1	KOB74596.1	*Operophtera brumata*	Lepidoptera
MbCDA1	AEI30868.1	*Mamestra brassicae*	Lepidoptera
TcCDA1	NP_001095946.1	*Tribolium castaneum*	Coleoptera
CmCDA4	AJG44548.1	*Cnaphalocrocis medinalis*	Lepidoptera
HvCDA4	QDZ05990.1	*Heortia vitessoides*	Lepidoptera
TcCDA4	NP_001103903.1	*Tribolium castaneum*	Coleoptera
LdCDA4	AWX65385.1	*Leptinotarsa decemlineata*	Coleoptera
CpCHT1	ASM94206.1	*Conogethes punctiferalis*	Lepidoptera
BmCHT1	NP_001166831.1	*Bombyx mori*	Lepidoptera
MsCHT-h	ABB88891.1	*Manduca sexta*	Lepidoptera
PxyCHT-h	AZS52299.1	*Plutella xylostella*	Lepidoptera
BmCHT-h	NP_001108407.1	*Bombyx mori*	Lepidoptera
OfCHT-h	BAE16587.1	*Ostrinia furnacalis*	Lepidoptera
CpCHT7	ASM94207.1	*Conogethes punctiferalis*	Lepidoptera
SeCHT7	AFM38213.1	*Spodoptera exigua*	Lepidoptera
TcCHT7	NP_001036035.1	*Tribolium castaneum*	Coleoptera
SlCHT2	XP_022837417.1	*Spodoptera litura*	Lepidoptera
PxuCHT2	KPI97752.1	*Papilio xuthus*	Lepidoptera
CsCHT2	ALO79339.1	*Chilo suppressalis*	Lepidoptera
DmCHT2	NP_477298.2	*Drosophila melanogaster*	Diptera
CmCHT2	AJG44542.1	*Cnaphalocrocis medinalis*	Lepidoptera
OfCHT2	XP_028167467.1	*Ostrinia furnacalis*	Lepidoptera
PxyCHT2	AZS52289.1	*Plutella xylostella*	Lepidoptera
BmCHT2	XP_004933352.2	*Bombyx mori*	Lepidoptera
HaCHT2	XP_021180780.1	*Helicoverpa armigera*	Lepidoptera
TcCHT2	XP_970191.2	*Tribolium castaneum*	Coleoptera
PpCHT3	XP_013135894.1	*Papilio polytes*	Lepidoptera
PxuCHT3	KPI96666.1	*Papilio xuthus*	Lepidoptera
AtCHT3	XP_013183423.1	*Amyelois transitella*	Lepidoptera
DmCHSB	NP_524209.3	*Drosophila melanogaster*	Diptera
DmCHT7	NP_647768.3	*Drosophila melanogaster*	Diptera

Table S3. The detail information of genes involved in detoxification in *G. Pyloalis*.

Gene ID	Inte rpkm	Midg rpkm	Larv rpkm	Description	Species
**CYP**					
Unigene0000060	4.8	29.7	13.1	cytochrome P450	*Cnaphalocrocis medinalis*
Unigene0001364	7.5	6.0	15.9	cytochrome P450 monooxygenase CYP18A1	*Cnaphalocrocis medinalis*
Unigene0001382	1.2	70.9	30.6	cytochrome P450 monooxygenase CYP6AE76	*Cnaphalocrocis medinalis*
Unigene0003810	33.6	0.7	8.2	cytochrome P450 monooxygenase CYP4G112	*Cnaphalocrocis medinalis*
Unigene0004694	2.8	10.5	4.0	cytochrome P450 monooxygenase CYP306A1, partial	*Cnaphalocrocis medinalis*
Unigene0005129	74.7	8.1	410.8	cytochrome P450 monooxygenase CYP304F17, partial	*Cnaphalocrocis medinalis*
Unigene0006921	13.8	18.3	23.3	cytochrome P450 monooxygenase CYP9G19	*Cnaphalocrocis medinalis*
Unigene0010223	19.6	8.2	10.3	cytochrome P450 monooxygenase CYP333A13	*Cnaphalocrocis medinalis*
Unigene0013997	4.1	0.0	3.2	cytochrome P450 monooxygenase CYP367B12	*Cnaphalocrocis medinalis*
Unigene0014581	0.3	0.1	0.8	cytochrome P450 monooxygenase CYP4CG17	*Cnaphalocrocis medinalis*
Unigene0015906	0.9	0.5	3.0	cytochrome P450 monooxygenase CYP321C7	*Cnaphalocrocis medinalis*
Unigene0019755	0.9	0.5	3.4	cytochrome P450 monooxygenase CYP337B12	*Cnaphalocrocis medinalis*
Unigene0021416	0.1	0.7	0.5	cytochrome P450 monooxygenase CYP4M25	*Cnaphalocrocis medinalis*
Unigene0025384	0.5	15.5	5.1	cytochrome P450 monooxygenase CYP333A13	*Cnaphalocrocis medinalis*
Unigene0025593	59.6	18.5	37.6	cytochrome P450 monooxygenase CYP9G18	*Cnaphalocrocis medinalis*
Unigene0026095	1.6	19.2	11.6	cytochrome P450 monooxygenase CYP304F17, partial	*Cnaphalocrocis medinalis*
Unigene0026795	4.0	18.1	13.8	cytochrome P450 monooxygenase CYP324A19	*Cnaphalocrocis medinalis*
Unigene0027351	1.1	0.6	1.9	cytochrome P450 monooxygenase CYP333B27	*Cnaphalocrocis medinalis*
Unigene0028612	3.3	0.1	21.2	cytochrome P450 monooxygenase CYP4G113	*Cnaphalocrocis medinalis*
Unigene0029457	14.6	0.8	5.6	cytochrome P450 monooxygenase CYP301A1	*Cnaphalocrocis medinalis*
Unigene0029599	2.5	1.9	2.1	cytochrome P450 monooxygenase CYP305B1	*Cnaphalocrocis medinalis*

Unigene0030964	6.3	1.0	1.6	cytochrome P450 monooxygenase CYP9A80	*Cnaphalocrocis medinalis*
Unigene0031583	9.2	24.8	13.0	cytochrome P450 monooxygenase CYP333B28	*Cnaphalocrocis medinalis*
Unigene0032126	0.7	5.4	3.5	cytochrome P450 monooxygenase CYP6CV1	*Cnaphalocrocis medinalis*
Unigene0032150	0.9	8.6	2.8	cytochrome P450 monooxygenase CYP6AW1	*Cnaphalocrocis medinalis*
Unigene0032238	0.6	0.1	6.9	cytochrome P450 monooxygenase CYP339A1	*Cnaphalocrocis medinalis*
Unigene0032568	1.2	3.2	2.0	cytochrome P450 monooxygenase CYP9A79	*Cnaphalocrocis medinalis*
Unigene0036420	3.3	0.0	1.1	cytochrome P450 monooxygenase CYP307A2	*Cnaphalocrocis medinalis*
Unigene0036473	3.0	1.1	14.0	cytochrome P450 monooxygenase CYP321F5	*Cnaphalocrocis medinalis*
Unigene0024333	0.5	0.5	0.2	cytochrome P450 2A4	*Mus musculus*
Unigene0024627	0.6	0.5	0.1	cytochrome P450 2J5 [Mus musculus]	*Mus musculus*
Unigene0002530	1.8	1.3	0.5	cytochrome P450 4B1 [Mus musculus]	*Mus musculus*
Unigene0010875	0.7	0.4	0.2	cytochrome P450 2A5 [Mus musculus]	*Mus musculus*
Unigene0027010	0.4	0.5	0.2	cytochrome P450 2D9 [Mus musculus]	*Mus musculus*
Unigene0027011	0.3	0.1	0.0	PREDICTED: cytochrome P450 2D26 isoform X1	*Mus musculus*
Unigene0008415	0.1	0.2	0.0	cytochrome P450 4A10	*Mus musculus*
Unigene0008669	0.3	0.2	0.0	cytochrome P450 4A14	*Mus musculus*
Unigene0008670	0.3	0.1	0.2	PREDICTED: cytochrome P450 4A14 isoform X1	*Mus musculus*
Unigene0010460	0.3	0.1	0.1	cytochrome P450 3A11	*Mus musculus*
Unigene0014391	0.3	0.1	0.1	PREDICTED: cytochrome P450, family 4, subfamily a, polypeptide 32 isoform X4	*Mus musculus*
Unigene0015627	1.4	1.4	0.6	cytochrome P450 2E1	*Mus musculus*
Unigene0018236	0.3	0.3	0.1	cytochrome P450, family 2, subfamily f, polypeptide 2, isoform CRA_c	*Mus musculus*
Unigene0018237	0.3	0.4	0.1	cytochrome P-450 naphthalene hydroxylase	*Mus musculus*
Unigene0022714	0.3	0.4	0.2	Cytochrome P450, family 3, subfamily a, polypeptide 11	*Mus musculus*
Unigene0005207	0.6	0.1	0.1	CYP302A1, partial	*Chilo suppressalis*
Unigene0007250	1.0	0.0	0.3	CYP341B10, partial	*Chilo suppressalis*

Unigene0008057	0.1	0.0	1.0	CYP6CT1	*Chilo suppressalis*
Unigene0008058	0.1	0.0	0.9	CYP6CT1	*Chilo suppressalis*
Unigene0008277	0.1	0.0	0.3	CYP6CT1	*Chilo suppressalis*
Unigene0019102	2.5	52.3	19.1	CYP6AB51	*Chilo suppressalis*
Unigene0019104	1.7	0.7	4.0	CYP6AB51	*Chilo suppressalis*
Unigene0020459	0.0	0.1	0.2	CYP6AB46	*Chilo suppressalis*
Unigene0025393	0.2	5.9	2.3	CYP6AB49	*Chilo suppressalis*
Unigene0027137	0.3	23.6	6.6	CYP6AB49	*Chilo suppressalis*
Unigene0027949	0.2	0.3	1.2	CYP6AB52	*Chilo suppressalis*
Unigene0033059	2.6	11.7	9.6	CYP6AB51	*Chilo suppressalis*
Unigene0033700	6.6	3.8	2.8	CYP6AB47	*Chilo suppressalis*
Unigene0000516	0.0	9.7	1.9	cytochrome P450 CYP6AB10	*Helicoverpa armigera*
Unigene0020508	0.4	0.2	1.1	cytochrome P450 CYP341B2	*Helicoverpa armigera*
Unigene0027009	1.9	36.3	10.7	cytochrome P450 CYP6AB10	*Helicoverpa armigera*
Unigene0035382	7881.2	7535.2	4631.4	cytochrome P450-like TBP, partial	*Helicoverpa armigera*
Unigene0028641	10.7	1.8	7.7	cytochrome P450 CYP49A1	*Helicoverpa armigera*
Unigene0030040	5.6	0.2	1.7	cytochrome P450 CYP4AU1	*Helicoverpa armigera*
Unigene0029479	7.5	0.3	2.2	cytochrome P450 CYP4AU1	*Helicoverpa armigera*
Unigene0037037	8.5	0.0	2.0	cytochrome P450 CYP341B2	*Helicoverpa armigera*
Unigene0001714	5.2	0.0	1.4	CYP303A1	*Helicoverpa armigera*
Unigene0030501	6.2	0.1	1.9	PREDICTED: cytochrome P450 4C1-like	*Amyelois transitella*
Unigene0006682	0.0	0.4	0.3	PREDICTED: cytochrome P450 6B2-like	*Amyelois transitella*
Unigene0005281	1.9	4.1	2.2	PREDICTED: cytochrome P450 9e2-like	*Amyelois transitella*
Unigene0005414	0.1	0.1	0.9	PREDICTED: probable cytochrome P450 4ac1	*Amyelois transitella*
Unigene0036027	2.2	0.0	1.1	PREDICTED: probable cytochrome P450 49a1	*Amyelois transitella*
Unigene0031389	10.4	1.9	8.9	PREDICTED: cytochrome P450 4c3	*Bombyx mori*

Unigene0018644	0.1	0.3	0.3	PREDICTED: cytochrome P450 9e2-like	*Bombyx mori*
Unigene0024232	0.1	0.1	0.5	PREDICTED: cytochrome P450 9e2-like	*Bombyx mori*
Unigene0029458	1.5	5.0	4.5	cytochrome P450, partial	*Bombyx mori*
Unigene0005526	0.1	0.5	0.3	Cytochrome P450 protein, partial	*Operophtera brumata*
Unigene0008286	0.1	0.1	0.2	Cytochrome P450 protein, partial	*Operophtera brumata*
Unigene0012956	0.5	0.2	0.2	Cytochrome P450 protein, partial	*Operophtera brumata*
Unigene0015860	0.2	0.1	0.0	Cytochrome P450 protein, partial	*Operophtera brumata*
Unigene0016566	0.4	0.5	0.3	Cytochrome P450 protein, partial	*Operophtera brumata*
Unigene0022769	0.2	0.1	0.4	Cytochrome P450 protein, partial	*Operophtera brumata*
Unigene0023966	0.2	0.3	0.3	Cytochrome P450 protein, partial	*Operophtera brumata*
Unigene0018024	5.2	1.5	6.6	PREDICTED: probable cytochrome P450 6a23	*Papilio xuthus*
Unigene0005415	0.0	0.0	1.0	Cytochrome P450 4C1	*Papilio xuthus*
Unigene0020702	0.1	0.2	0.3	PREDICTED: cytochrome P450 84A4	*Rhinolophus sinicus*
Unigene0022458	0.0	0.0	0.5	PREDICTED: cytochrome P450 81D11-like, partial	*Rhinolophus sinicus*
Unigene0023670	0.2	0.1	0.1	PREDICTED: cytochrome P450 81F2, partial	*Rhinolophus sinicus*
Unigene0029865	6.1	0.4	2.1	DIMBOA-induced cytochrome P450	*Ostrinia furnacalis*
Unigene0001070	0.5	0.2	1.3	PREDICTED: cytochrome P450 4c3-like	*Papilio machaon*
Unigene0010525	0.2	0.0	0.7	Cytochrome P450 4C1	*Papilio machaon*
Unigene0005208	0.7	0.0	0.2	cytochrome P450 CYP302A1	*Manduca sexta*
Unigene0025865	1.0	8.9	4.8	PREDICTED: cytochrome P450 6k1-like	*Plutella xylostella*
**GST**					
Unigene0023789	143.1	167.8	120.3	microsomal glutathione S-transferase 1	*Pararge aegeria*
Unigene0014508	0.6	0.4	0.7	glutathione S-transferase delta 1	*Cnaphalocrocis medinalis*
Unigene0007820	127.3	173.4	143.9	glutathione S-transferase omega 1	*Cnaphalocrocis medinalis*
Unigene0006385	5.4	6.2	4.4	PREDICTED: glutathione S-transferase theta-1-like	*Papilio xuthus*
Unigene0006800	0.1	0.0	1.8	microsomal glutathione S-transferase 1-5	*Spodoptera litura*

Unigene0006801	0.0	0.0	1.0	microsomal glutathione S-transferase 1-5	*Spodoptera litura*
Unigene0016557	0.3	0.0	0.0	PREDICTED: glutathione S-transferase 1 isoform X4	*Harpegnathos saltator*
Unigene0019530	1.3	1.6	0.6	microsomal glutathione S-transferase 1 isoform 2	*[Mus musculus*
Unigene0028029	0.1	0.0	2.7	microsomal glutathione S-transferase 1-4	*Spodoptera litura*
Unigene0028232	18.8	5.6	7.5	glutathione S-transferase zeta 1	*Chilo suppressalis*
Unigene0001415	15.7	0.1	4.1	glutathione S-transferase epsilon 2	*Chilo suppressalis*
Unigene0017940	3.0	4.4	3.0	glutathione S-transferase omega 2	*Chilo suppressalis*
Unigene0026383	0.0	10.4	3.5	glutathione S-transferase epsilon 2	*Bombyx mori*
Unigene0027760	15.8	45.8	35.0	glutathione S-transferase omega 2	*Cnaphalocrocis medinalis*
Unigene0028294	1.9	10.2	5.0	glutathione S-transferase epsilon 2	*Cnaphalocrocis medinalis*
Unigene0030164	1070.0	600.8	598.6	glutathione S-transferase delta 2	*Cnaphalocrocis medinalis*
Unigene0035664	4.1	6.0	4.8	glutathione S-transferase zeta 2	*Cnaphalocrocis medinalis*
Unigene0011567	7.1	56.5	36.4	glutathione S-transferase epsilon 3	*Cnaphalocrocis medinalis*
Unigene0018590	1687.4	954.1	6628.0	glutathione S-transferase sigma 3	*Cnaphalocrocis medinalis*
Unigene0026633	14.0	11.1	8.6	glutathione S-transferase omega 3	*Cnaphalocrocis medinalis*
Unigene0028828	228.2	130.6	847.5	glutathione S-transferase sigma 4	*Cnaphalocrocis medinalis*
Unigene0030256	3.7	20.3	15.1	glutathione S-transferase epsilon 4	*Cnaphalocrocis medinalis*
Unigene0008315	122.1	404.2	258.6	glutathione S-transferase sigma 5	*Cnaphalocrocis medinalis*
Unigene0023681	1.0	0.0	0.4	glutathione S-transferase epsilon 5, partial	*Cnaphalocrocis medinalis*
Unigene0011019	0.2	0.3	0.7	glutathione S-transferase III homolog	*Naegleria fowleri*
Unigene0015218	39.5	1.1	8.1	glutathione S-transferase	*Cnaphalocrocis medinalis*
Unigene0033982	9.9	8.0	7.5	glutathione S-transferase	*Cnaphalocrocis medinalis*
Unigene0015848	0.4	0.1	0.0	PREDICTED: probable glutathione S-transferase	*Aplysia californica*
Unigene0016824	44.3	70.5	44.5	microsomal glutathione S-transferase	*Antheraea yamamai*
Unigene0024138	0.8	0.9	0.3	glutathione S-transferase P 1	*Mus musculus*
Unigene0026170	0.8	0.7	0.2	glutathione S-transferase A3 isoform a	*Mus musculus*

Unigene0019555	1.7	1.3	1.0	PREDICTED: glutathione S-transferase C-terminal domain-containing protein homolog	*Amyelois transitella*
Unigene0003982	5.8	50.2	13.5	PREDICTED: glutathione S-transferase-like	*Amyelois transitella*
Unigene0034170	28.4	19.3	26.3	glutathione S-transferase	*Ostrinia furnacalis*
Unigene0036070	176.6	62.4	807.4	glutathione S-transferase	*Choristoneura fumiferana*
Unigene0015760	0.4	0.7	6.3	glutathione S-transferase epsilon 8	*Cnaphalocrocis medinalis*
Unigene0027235	4.2	8.3	5.7	glutathione S-transferase epsilon 9	*Cnaphalocrocis medinalis*
				**CarE**	
Unigene0003686	13.6	1.8	9.1	carboxylesterase	*Cnaphalocrocis medinalis*
Unigene0008425	7.0	10.8	6.6	carboxylesterase	*Cnaphalocrocis medinalis*
Unigene0009737	84.4	0.8	24.5	carboxylesterase	*Cnaphalocrocis medinalis*
Unigene0010160	33.6	54.1	37.6	carboxylesterase	*Cnaphalocrocis medinalis*
Unigene0010161	0.7	6.1	3.4	carboxylesterase	*Cnaphalocrocis medinalis*
Unigene0010592	0.3	5.9	1.7	carboxylesterase	*Cnaphalocrocis medinalis*
Unigene0014627	8.9	10.8	7.8	carboxylesterase	*Cnaphalocrocis medinalis*
Unigene0015365	6.0	28.8	20.3	carboxylesterase	*Cnaphalocrocis medinalis*
Unigene0018985	24.9	68.7	49.2	carboxylesterase	*Cnaphalocrocis medinalis*
Unigene0019725	84.7	313.6	110.5	carboxylesterase	*Cnaphalocrocis medinalis*
Unigene0025903	34.9	11.6	40.4	carboxylesterase	*Cnaphalocrocis medinalis*
Unigene0028596	1.4	2.3	1.1	carboxylesterase	*Cnaphalocrocis medinalis*
Unigene0031141	140.8	40.2	138.0	carboxylesterase	*Cnaphalocrocis medinalis*
Unigene0034810	1.7	27.8	10.4	carboxylesterase	*Cnaphalocrocis medinalis*
Unigene0035728	0.0	1.3	1.1	carboxylesterase	*Cnaphalocrocis medinalis*
Unigene0011498	0.3	0.5	0.0	PREDICTED: carboxylesterase 3A isoform X1	*Mus musculus*
Unigene0012778	0.1	0.2	0.0	PREDICTED: carboxylesterase 3A isoform X2	*Mus musculus*
Unigene0012779	0.1	0.1	0.0	PREDICTED: carboxylesterase 3A isoform X2	*Mus musculus*

Unigene0032082	0.4	0.5	0.2	carboxylesterase 1C precursor	*Mus musculus*
Unigene0032083	0.4	0.4	0.2	carboxylesterase 1F precursor	*Mus musculus*
Unigene0032084	0.3	0.2	0.0	PREDICTED: liver carboxylesterase B-1 isoform X1	*Mus musculus*
Unigene0003837	5.5	0.2	2.1	carboxylesterase	*Ostrinia furnacalis*
Unigene0013473	5.4	13.1	5.5	carboxylesterase	*Ostrinia furnacalis*
Unigene0014261	6.4	1.9	2.9	carboxylesterase	*Ostrinia furnacalis*
Unigene0022777	0.3	0.2	1.7	carboxylesterase	*Ostrinia furnacalis*
Unigene0023257	1.0	4.2	3.0	carboxylesterase	*Ostrinia furnacalis*
Unigene0032549	0.6	0.2	0.1	carboxylesterase, partial	*Ostrinia furnacalis*
Unigene0035729	0.1	1.6	0.5	carboxylesterase, partial	*Ostrinia furnacalis*
Unigene0036228	1.9	1.9	2.5	carboxylesterase	*Ostrinia furnacalis*
Unigene0008442	0.0	25.9	4.7	Carboxylesterase-like protein, partial	*Operophtera brumata*
Unigene0008718	0.2	0.3	0.4	Carboxylesterase CXE23, partial	*Operophtera brumata*
Unigene0008927	0.1	128.6	28.8	Carboxylesterase-like protein, partial	*Operophtera brumata*
Unigene0001354	0.2	0.0	0.0	Carboxylesterase CXE23, partial	*Operophtera brumata*
Unigene0005121	0.2	0.0	0.0	Carboxylesterase CXE23, partial	*Operophtera brumata*
Unigene0008191	0.3	0.3	0.1	Carboxylesterase CXE23, partial	*Operophtera brumata*
Unigene0014983	0.2	0.2	0.4	Carboxylesterase CXE23, partial	*Operophtera brumata*
Unigene0018466	0.0	0.4	0.1	Carboxylesterase CXE23, partial	*Operophtera brumata*
Unigene0021969	0.3	0.0	0.0	Carboxylesterase CXE23, partial	*Operophtera brumata*
Unigene0024017	0.1	0.2	0.1	Carboxylesterase CXE23, partial	*Operophtera brumata*
Unigene0021634	0.1	0.6	0.3	antennal carboxylesterase 15, partial	*Chilo suppressalis*
Unigene0008401	25.6	295.2	148.7	carboxylesterase	*Chilo suppressalis*
Unigene0005442	3.9	6.0	39.6	antennal carboxylesterase 15, partial	*Chilo suppressalis*
Unigene0005766	0.1	0.7	0.5	antennal carboxylesterase 4, partial	*Chilo suppressalis*
Unigene0000629	361.3	3.3	108.3	PREDICTED: carboxylesterase 1E	*Amyelois transitella*

Unigene0028666	55.9	0.0	8.2	PREDICTED: carboxylesterase 1E	*Amyelois transitella*
Unigene0031362	0.8	5.4	2.3	PREDICTED: palmitoleoyl-protein carboxylesterase NOTUM	*Amyelois transitella*
Unigene0028549	38.8	2.6	54.3	Carboxylesterase 4A	*Papilio xuthus*
Unigene0033927	1.3	0.4	0.6	PREDICTED: venom carboxylesterase-6-like	*Papilio xuthus*
Unigene0034570	0.1	0.1	0.2	PREDICTED: carboxylesterase 1C isoform X1	*Papilio xuthus*
Unigene0035671	4.1	0.0	0.6	Liver carboxylesterase	*Papilio xuthus*
Unigene0005827	0.7	0.1	1.3	PREDICTED: venom carboxylesterase-6-like	*Plutella xylostella*
Unigene0028217	3.4	0.1	9.3	PREDICTED: venom carboxylesterase-6-like	*Plutella xylostella*
Unigene0015075	1.4	0.4	3.2	carboxylesterase 6	*Plutella xylostella*
Unigene0018075	3.5	46.0	17.1	carboxylesterase	*Helicoverpa armigera*
Unigene0028584	0.1	31.2	11.9	carboxylesterase	*Helicoverpa armigera*
Unigene0035791	1.7	0.1	0.3	carboxylesterase	*Bombyx mandarina*
Unigene0035792	0.9	0.0	0.0	carboxylesterase	*Bombyx mandarina*
Unigene0026451	0.1	18.9	5.9	carboxylesterase CarE-11 precursor	*Bombyx mori*
Unigene0034568	0.4	0.1	0.2	Carboxylesterase 4A	*Papilio machaon*
**Nicotinic acetylcholine receptor**					
Unigene0004400	0.7	0.7	0.6	nicotinic acetylcholine receptor alpha 3 subunit	*Chilo suppressalis*
Unigene0009747	2.8	0.0	0.7	nicotinic acetylcholine receptor alpha 7 subunit variant 1	*Chilo suppressalis*
Unigene0011898	0.4	0.0	0.5	nicotinic acetylcholine receptor alpha 8 subunit variant 1	*Chilo suppressalis*
Unigene0013009	8.5	0.3	24.4	nicotinic acetylcholine receptor alpha 9 subunit	*Chilo suppressalis*
Unigene0018844	0.5	0.1	0.0	nicotinic acetylcholine receptor alpha subunit precursor	*Chilo suppressalis*
Unigene0019713	2.1	0.1	7.7	nicotinic acetylcholine receptor alpha 9 subunit	*Chilo suppressalis*
Unigene0023735	2.6	0.1	9.7	nicotinic acetylcholine receptor beta 3 subunit	*Chilo suppressalis*
**Aquaporin**					
Unigene0009112	0.0	0.1	0.2	Aquaporin TIP3-2	*Trichinella papuae*
Unigene0009113	0.2	0.0	0.2	Aquaporin TIP3-2	*Trichinella papuae*

Unigene0009114	0.4	0.3	0.8	Aquaporin TIP3-2	*Trichinella papuae*
Unigene0015427	0.8	3.2	2.6	PREDICTED: aquaporin AQPAn.G-like isoform X2	*Amyelois transitella*
Unigene0016245	93.5	31.9	154.6	PREDICTED: aquaporin AQPAe.a-like	*Bombyx mori*
Unigene0019115	0.0	0.2	0.0	PREDICTED: probable aquaporin PIP1-5	*Rhinolophus sinicus*
Unigene0024324	0.5	0.4	0.2	aquaporin-1	*Mus musculus*
Unigene0025307	0.6	22.6	6.7	PREDICTED: aquaporin-like	*Amyelois transitella*
Unigene0026164	73.8	51.1	46.2	aquaporin-1 variant A	*Chilo suppressalis*
Unigene0026716	0.1	15.0	4.2	PREDICTED: aquaporin-like	*Amyelois transitella*
Unigene0027202	82.1	8.8	17.9	PREDICTED: aquaporin-12 isoform X1	*Amyelois transitella*
Unigene0033913	0.2	0.4	0.9	Aquaporin TIP3-2	*Trichinella papuae*
Unigene0033914	0.2	0.6	0.4	Aquaporin TIP3-2	*Trichinella papuae*
Unigene0034032	0.4	0.1	0.2	PREDICTED: aquaporin PIP2-7 isoform X2	*Amyelois transitella*
Unigene0034033	0.4	0.4	0.2	PREDICTED: aquaporin PIP2-7 isoform X2	*Amyelois transitella*
Unigene0036454	0.5	0.1	0.7	PREDICTED: aquaporin PIP1-2	*Rhinolophus sinicus*
Unigene0036455	0.4	0.1	0.5	PREDICTED: aquaporin PIP2-1	*Rhinolophus sinicus*
Unigene0036457	0.1	0.0	0.1	PREDICTED: aquaporin PIP1-3	*Rhinolophus sinicus*
Unigene0036458	0.1	0.3	0.6	PREDICTED: aquaporin PIP1-2	*Rhinolophus sinicus*
**chloride channel**					
Unigene0003997	3.8	11.6	6.1	PREDICTED: chloride channel protein 2 isoform X2	*Papilio xuthus*
Unigene0006250	0.5	0.0	0.0	PREDICTED: glutamate-gated chloride channel	*Bombyx mori*
Unigene0025368	1.7	0.1	0.5	Glutamate-gated chloride channel	*Papilio xuthus*
**Methoprene-tolerant**					
Unigene0002198	0.4	0.1	0.2	methoprene-tolerant protein, partial	*Omphisa fuscidentalis*
Unigene0030821	2.5	1.7	1.9	juvenile hormone receptor methoprene-tolerant	*Plodia interpunctella*
**Serine protease**					
Unigene0002596	1.5	1.3	5.6	serine protease inhibitor 28	*Danaus plexippus*

Unigene0003070	1.1	0.3	0.3	PREDICTED: serine protease inhibitor 34 isoform X1	*Bombyx mori*
Unigene0003290	7.8	0.0	1.2	serine protease inhibitor 001	*Chilo suppressalis*
Unigene0005999	31.9	0.6	6.8	serine protease inhibitor 002	*Chilo suppressalis*
Unigene0006041	12.8	9.7	26.4	serine protease inhibitor 012	*Chilo suppressalis*
Unigene0007292	68.1	11.0	24.4	serine protease inhibitor 006	*Chilo suppressalis*
Unigene0010733	45.5	4.9	62.8	serine protease inhibitor 003	*Chilo suppressalis*
Unigene0012077	6.8	7.2	6.0	serine protease inhibitor 013	*Chilo suppressalis*
Unigene0015234	0.0	1.1	0.3	PREDICTED: kunitz serine protease inhibitor Pr-mulgin 2-like	*Bombyx mori*
Unigene0019762	48.9	2.5	8.4	PREDICTED: serine protease inhibitor dipetalogastin-like, partial	*Amyelois transitella*
Unigene0021134	0.6	1.2	0.2	serine protease inhibitor A3K precursor	*Mus musculus*
Unigene0021591	35.6	0.7	11.2	serine protease inhibitor 008	*Chilo suppressalis*
Unigene0025153	6.9	68.3	30.3	serine protease inhibitor 012	*Chilo suppressalis*
Unigene0025901	4.6	0.8	8.4	serine protease inhibitor	*Danaus plexippus*
Unigene0026251	71.1	0.7	15.5	PREDICTED: serine protease inhibitor 3/4	*Amyelois transitella*
Unigene0026506	85.6	13.1	56.7	serine protease inhibitor 3	*Ostrinia furnacalis*
Unigene0027248	0.0	0.0	1.0	PREDICTED: kunitz-type serine protease inhibitor Bt-KTI-like	*Dufourea novaeangliae*
Unigene0029163	12.6	2.3	9.6	serine protease inhibitor 010	*Chilo suppressalis*
Unigene0031118	3.3	0.3	8.8	serine protease inhibitor 5	*Helicoverpa armigera*
Unigene0031825	67.1	11.2	222.6	PREDICTED: serine protease inhibitor 2.1-like	*Papilio machaon*
Unigene0035936	0.1	6.9	15.4	serine protease inhibitor 012	*Chilo suppressalis*
Unigene0036936	0.8	0.0	0.0	PREDICTED: serine protease inhibitor 3/4-like isoform X14	*Polistes canadensis*
**Sodium channel**					
Unigene0008421	0.5	0.2	0.5	PREDICTED: sodium channel protein Nach-like	*Amyelois transitella*
Unigene0008422	0.3	0.1	0.4	PREDICTED: sodium channel protein Nach-like	*Amyelois transitella*
Unigene0008423	0.2	0.0	0.2	PREDICTED: sodium channel protein Nach-like	*Amyelois transitella*
Unigene0010090	0.2	0.0	0.5	PREDICTED: sodium channel protein Nach-like	*Amyelois transitella*

Unigene0010466	0.0	0.0	0.3	PREDICTED: sodium channel protein Nach-like	*Amyelois transitella*
Unigene0014046	1.3	0.1	0.3	voltage-gated sodium channel alpha subunit	*Bombyx mori*
Unigene0022621	0.0	0.6	0.1	PREDICTED: sodium channel protein Nach-like	*Bombyx mori*
Unigene0029785	0.3	0.0	0.2	Sodium channel protein, partial	*Operophtera brumata*
Unigene0029787	0.7	0.2	0.9	Sodium channel protein 60E	*Papilio xuthus*
Unigene0029788	0.5	0.2	0.7	PREDICTED: sodium channel protein 60E-like	*Amyelois transitella*
Unigene0029791	0.2	0.1	0.7	PREDICTED: sodium channel protein 60E-like	*Papilio xuthus*
**Calcium channel**					
Unigene0002803	6.7	0.4	1.6	PREDICTED: muscle calcium channel subunit alpha-1-like isoform X1	*Papilio xuthus*
Unigene0003833	0.4	0.0	0.1	PREDICTED: muscle calcium channel subunit alpha-1-like isoform X6	*Papilio xuthus*
Unigene0003834	0.7	0.0	0.3	PREDICTED: muscle calcium channel subunit alpha-1-like	*Amyelois transitella*
Unigene0004983	0.5	0.0	0.1	PREDICTED: muscle calcium channel subunit alpha-1-like	*Amyelois transitella*
Unigene0004984	0.6	0.0	0.2	Voltage-dependent calcium channel type D subunit alpha-1	*Papilio xuthus*
Unigene0005032	0.1	0.3	0.3	PREDICTED: voltage-dependent calcium channel subunit alpha-2/delta-3-like	*Papilio polytes*
Unigene0005034	0.2	1.2	0.4	PREDICTED: voltage-dependent calcium channel subunit alpha-2/delta-4-like	*Papilio xuthus*
Unigene0005036	0.1	1.2	0.5	PREDICTED: voltage-dependent calcium channel subunit alpha-2/delta-4-like	*Papilio machaon*
Unigene0005482	1.0	3.8	2.2	PREDICTED: two pore calcium channel protein 2-like isoform X4	*Papilio xuthus*
Unigene0008120	0.4	0.1	0.0	PREDICTED: muscle calcium channel subunit alpha-1-like	*Bombyx mori*
Unigene0008121	0.6	0.0	0.2	PREDICTED: muscle calcium channel subunit alpha-1-like	*Amyelois transitella*
Unigene0009614	13.0	0.6	3.4	PREDICTED: voltage-dependent calcium channel gamma-4 subunit isoform X1	*Papilio machaon*
Unigene0010788	0.6	0.2	2.6	PREDICTED: voltage-dependent calcium channel subunit alpha-2/delta-3	*Amyelois transitella*
Unigene0015458	17.2	2.1	5.2	PREDICTED: voltage-dependent calcium channel subunit alpha-2/delta-4 isoform X1	*Amyelois transitella*
Unigene0026099	2.2	0.1	0.5	PREDICTED: voltage-dependent calcium channel subunit alpha-2/delta-3 isoform X5	*Papilio xuthus*
Unigene0026100	1.9	0.1	0.2	PREDICTED: voltage-dependent calcium channel subunit alpha-2/delta-3	*Amyelois transitella*

Unigene0026130	1.4	0.0	0.2	PREDICTED: voltage-dependent calcium channel type A subunit alpha-1	*Papilio polytes*
Unigene0028663	6.0	2.9	4.6	PREDICTED: calcium release-activated calcium channel protein 1-like isoform X2	*Amyelois transitella*
Unigene0029704	3.2	9.8	4.5	PREDICTED: calcium channel flower isoform X2	*Papilio xuthus*
Unigene0029864	1.9	2.2	1.1	Dihydropyridine-sensitive l-type calcium channel	*Operophtera brumata*
Unigene0034463	0.7	0.2	0.2	PREDICTED: muscle calcium channel subunit alpha-1-like	*Amyelois transitella*
Unigene0034966	1.0	0.7	0.8	PREDICTED: voltage-dependent T-type calcium channel subunit alpha-1G-like	*Amyelois transitella*
Unigene0034967	0.5	0.4	0.3	PREDICTED: voltage-dependent T-type calcium channel subunit alpha-1G-like	*Amyelois transitella*
Unigene0034968	0.2	0.2	0.3	PREDICTED: voltage-dependent T-type calcium channel subunit alpha-1G-like	*Papilio polytes*
Unigene0034969	0.2	0.3	0.1	PREDICTED: voltage-dependent T-type calcium channel subunit alpha-1G-like	*Amyelois transitella*
Unigene0034970	0.5	0.4	0.3	PREDICTED: voltage-dependent T-type calcium channel subunit alpha-1G-like	*Amyelois transitella*
Unigene0034971	0.3	0.5	0.1	PREDICTED: voltage-dependent T-type calcium channel subunit alpha-1G-like	*Plutella xylostella*
Unigene0035247	5.3	1.0	2.0	PREDICTED: voltage-dependent L-type calcium channel subunit beta-2 isoform X2	*Plutella xylostella*
Unigene0035248	6.6	1.2	1.8	Voltage-dependent L-type calcium channel subunit beta-2	*Papilio xuthus*
Unigene0036506	1.3	0.4	4.7	PREDICTED: voltage-dependent calcium channel subunit alpha-2/delta-4 isoform X2	*Papilio xuthus*
